# TERRA Promotes Telomere Shortening through Exonuclease 1–Mediated Resection of Chromosome Ends

**DOI:** 10.1371/journal.pgen.1002747

**Published:** 2012-06-14

**Authors:** Verena Pfeiffer, Joachim Lingner

**Affiliations:** Swiss Institute for Experimental Cancer Research (ISREC), School of Life Sciences, Frontiers in Genetics National Center of Competence in Research, Ecole Polytechnique Fédérale de Lausanne (EPFL), Lausanne, Switzerland; CEA/Fontenay, France

## Abstract

The long noncoding telomeric repeat containing RNA (TERRA) is expressed at chromosome ends. TERRA upregulation upon experimental manipulation or in ICF (immunodeficiency, centromeric instability, facial anomalies) patients correlates with short telomeres. To study the mechanism of telomere length control by TERRA in *Saccharomyces cerevisiae*, we mapped the transcriptional start site of TERRA at telomere 1L and inserted a doxycycline regulatable promoter upstream. Induction of TERRA transcription led to telomere shortening of 1L but not of other chromosome ends. TERRA interacts with the Exo1-inhibiting Ku70/80 complex, and deletion of *EXO1* but not *MRE11* fully suppressed the TERRA–mediated short telomere phenotype in presence and absence of telomerase. Thus TERRA transcription facilitates the 5′-3′ nuclease activity of Exo1 at chromosome ends, providing a means to regulate the telomere shortening rate. Thereby, telomere transcription can regulate cellular lifespan through modulation of chromosome end processing activities.

## Author Summary

Telomeres protect chromosome ends from end fusion and end degradation, and they regulate cellular lifespan. Telomerase, a reverse transcriptase, maintains telomere length. The end replication problem and the processing of DNA ends by nucleases cause telomere shortening. Telomeres are transcribed into a long noncoding RNA known as TERRA. ICF syndrome derived patient cells have short telomeres and enriched TERRA. TERRA inhibits telomerase activity *in vitro*. To analyze TERRA function *in vivo*, we used the model organism *Saccharomyces cerevisiae*. We identified the natural TERRA promoter at one chromosome end and replaced it with an artificial promoter to modulate TERRA transcription with a drug. TERRA induction leads to telomere shortening at this specific chromosome end. We show that telomere shortening is a consequence of increased activation of the 5′-3′ Exonuclease 1 at the transcribed telomere with no notable effects on telomerase recruitment and activity. The Ku protein controls Exonuclease 1 activity at chromosome ends. TERRA physically and genetically interacts with Ku and appears to interfere with its ability to inhibit Exonuclease 1. Thus, we demonstrate how TERRA expression controls telomere length through the regulation of the processing of chromosome ends. Therefore, telomere transcription can regulate cellular lifespan independently of telomerase.

## Introduction

The ends of eukaryotic chromosomes, known as telomeres, consist of simple DNA repeats, specialized proteins and long noncoding RNAs [1]. Telomeres protect chromosomes from degradation and end fusions [2]. In addition, telomere length regulates cellular lifespan in humans in which telomerase is turned off in most somatic cells [3]. Telomere length is a balance between the rate of telomere shortening, which occurs due to the end replication problem and the nucleolytic processing of chromosome ends and telomere elongation which is mediated by the cellular reverse transcriptase telomerase [4], [5], [6], [7], [8]. In *Saccharomyces cerevisiae*, telomere length homeostasis is achieved due to the telomere length dependent regulation of telomerase at individual chromosome ends. The likelihood that telomerase extends a telomere in a given cell cycle increases with telomere shortening [4]. The preferential recruitment and activation of telomerase to shorter telomeres is mediated by the Tel1 checkpoint kinase and Tbf1, a protein which binds to the subtelomeric DNA sequences present at natural chromosome ends [9], [10], [11], [12], [13]. On the other hand, the telomere shortening rate in the absence of telomerase is constant and appears length-independent [5].

Nucleolytic processing of chromosome ends can ensure that 3′ overhangs are present at all chromosome ends, independently of whether they are replicated by leading or lagging strand synthesis [7]. The single-stranded G-tails of budding yeast telomeres are short (about 10–15 nucleotides) for most of the cell cycle, and their length increases transiently in late S phase (to about 50–100 nucleotides) [14]. Recently, several activities have been identified that are involved in telomere DNA end processing [15]. Upon phosphorylation of Sae2 (CtIP in human) at Ser267 by Cdk1, this endonuclease in conjunction with the MRX complex (Mre11, Rad50, Xrs2) initiates 5′ end resection. The initial short telomeric 3′ overhang is further processed by the Sgs1-Dna2 helicase-5′-Flap endonuclease, but the 5′-3′ exonuclease Exo1 can also contribute to end resection. Exo1 activity at telomeres is restricted by the heterodimeric Ku complex (Ku70/80) as in its absence, telomeric ssDNA increases in an Exo1-dependent manner [16], [17], [18], [19]. Inactivation of Cdc13 also leads to accumulation of long ssDNA regions extending into non-telomeric DNA sequences due to Exo1 action [20], [21], [22].

TERRA is a long noncoding RNA which is expressed at chromosome ends of most or all eukaryotes [23]. TERRA acts as a potent mixed-type inhibitor of human telomerase *in vitro*, binding to both the RNA template and the TERT polypeptide [24], [25]. TERRA has also been proposed to mediate a switch of single strand telomere binding proteins at human telomeres after semiconservative DNA replication [26]. In the proposed model the single strand DNA binding protein RPA associates with telomeres during S phase of the cell cycle in order to promote semiconservative DNA replication. Upon replication, hnRNPA1 will transiently replace RPA at the G-strand overhang. This activity is inhibited by TERRA and therefore only occurs during S phase when TERRA levels, which are cell cycle regulated, decrease. Re-accumulation of TERRA after S phase leads to hnRNPA1 dissociation from the telomeric 3′overhang and allows finally POT1-TPP1 binding, which remains telomere-associated during the rest of the cell cycle. Finally, TERRA has been suspected to modulate telomeric chromatin structure, in analogy to other long noncoding RNAs [23].

Apart from its *in vitro* effects on telomerase, several genetic observations implicated TERRA in telomere length control. First, upregulation of TERRA at chromosome ends has been observed upon impairment with the nonsense-mediated mRNA decay machinery and this led to stochastic telomere loss of leading strand telomeres [27], [28]. Second, TERRA levels are also strongly increased in DNMT3b methyltransferase defective ICF-syndrome patient derived cells due to defects in subtelomeric DNA methylation. Also these cells have severely shortened telomeres [29]. Third, *rat1-1* mutant yeast cells, which have elevated TERRA levels carry shorter telomeres [30]. Fourth, Gal-promoter induced transcription of telomeric repeat DNA at a truncated chromosome lacking natural subtelomeric elements, led to telomere shortening [31]. To study the function and mechanism of TERRA for telomere length control at a natural telomere, we mapped the transcriptional start site of TERRA at telomere 1L to the X-core element, a repeat sequence found at all natural chromosome ends [32]. We inserted a regulatable promoter in order to study TERRA function for telomere length regulation. We find that TERRA transcription leads to telomere shortening *in cis*. TERRA transcription also accelerates the telomere shortening rate in the absence of telomerase. TERRA interacts with the Exo1-inhibiting Ku70/80 dimer and TERRA-mediated telomere shortening is alleviated by *EXO1* deletion in presence or absence of telomerase. Thus, TERRA transcription promotes Exo1-dependent resection at chromosome ends.

## Results

### TERRA start sites and development of an inducible system

**Figure 1:**
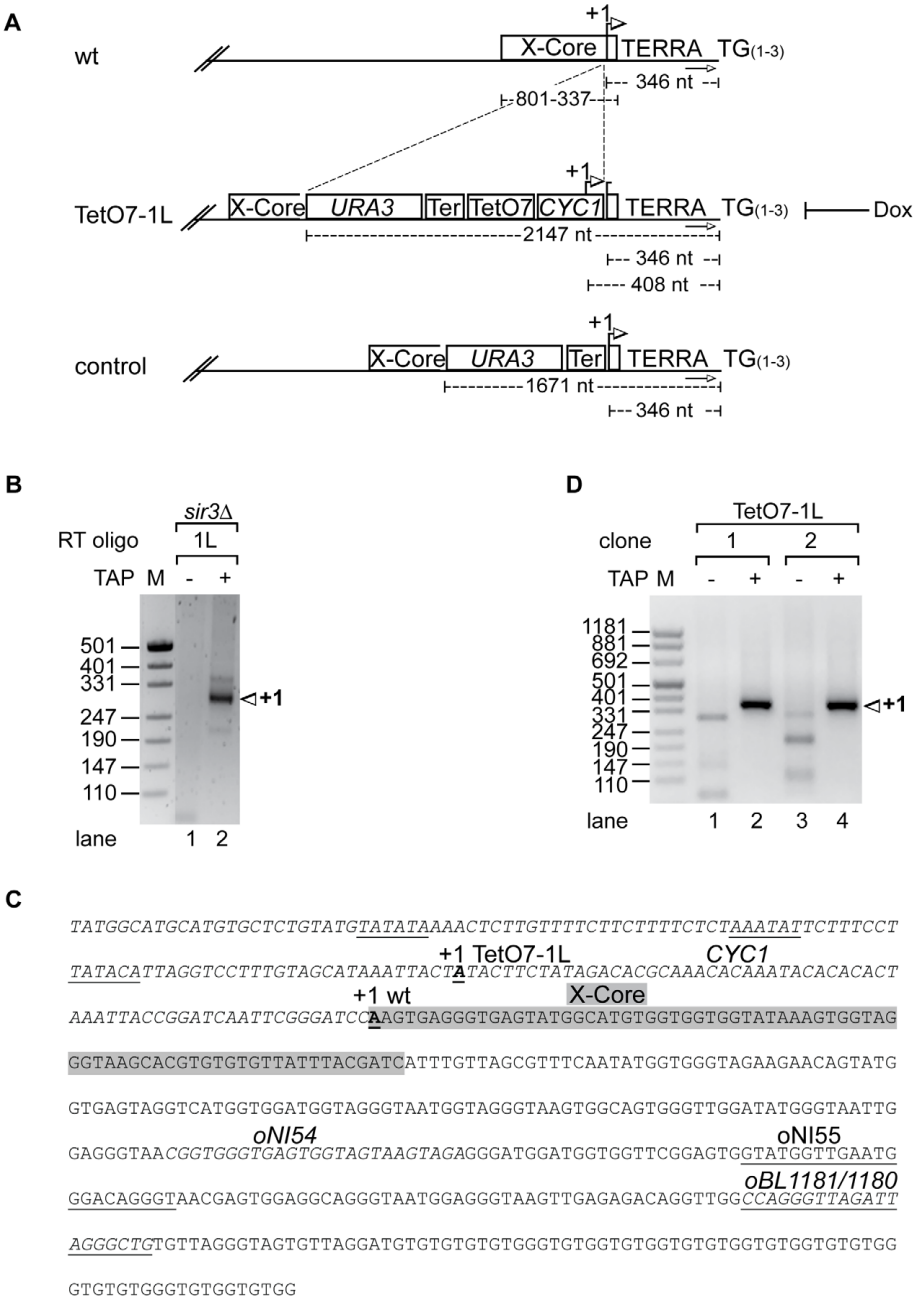
Development of an inducible 1L TERRA expression system. (A) Scheme of telomere 1L in wild type, the TetO7-1L and the control strains. +1 in the wt marks the transcription start site of 1L TERRA mapped in *sir3*Δ (see B, C). Numbers give the distance of the +1 site, the X-core element, or the *URA3* marker from the TG repeat sequence (TG_(1–3)_). The arrows towards the right mark the position of the subtelomeric oligonucleotide (oBL1180, see C) used for telomere PCR of telomere 1L. Strain TetO7-1L was constructed by introducing a sequence containing *URA3*, the *ADH1*-terminator (Ter), seven TetO boxes (TetO7) and a cytochrome 1 (*CYC1*) sequence upstream of the 1L TERRA transcription start site. +1 marks the transcription start site of the inducible 1L TERRA in strain TetO7-1L (see C, D). Presence of Dox inhibits 1L TERRA expression in TetO7-1L. The control strain was constructed by insertion of the *URA3* cassette and the *ADH1*-terminator upstream of the +1 start site, without the TetO7 and *CYC1* promoter sequences. (B) Nested PCR of 5′RACE (absence (−) or presence (+) of Tobacco Acid Pyrophosphatase (TAP): removes the 5′ cap structure of the RNA) performed in *sir3*Δ for mapping the transcription start site (+1) of 1L TERRA. Marker (M) is given in base pairs. (C) Sequence of telomere 1L in strain TetO7-1L. The *CYC1* sequence upstream of the +1 start site of 1L TERRA in wt (bold and underlined) is shown in italics. The putative TATA boxes of the *CYC1* sequence are underlined [44]. The +1 start site of 1L TERRA in TetO7-1L is shown in bold, and underlined. The X-core sequence is highlighted in grey. Marked are the oligonucleotides used for the RT of the 5′RACE (oBL1181; antisense strand), for telomere PCR of telomere 1L (oBL1180; sense strand), for the nested PCR (oNI55; antisense strand), and for 1L TERRA detection by qRT-PCR (oNI54 (sense strand), oBL1181) in the X-repeat sequence (downstream of the X-core sequence). (D) Nested PCR of 5′RACE (as in B) performed on two independently generated clones of strain TetO7-1L for mapping the transcriptional start site (+1) of 1L TERRA.

We mapped the transcriptional start site of TERRA at chromosome 1L (Figure 1A, 1B) by rapid amplification of 5′ ends (5′RACE) of capped RNAs in *S. cerevisiae* strain S288C *sir3Δ*. The 5′RACE was facilitated by deletion of *SIR3* as Sir3 represses telomere transcription [33]. 5′ RACE products were homogeneous in size (Figure 1B, lane 2) and cloning and sequencing of 5′ RACE products revealed existence of one major start site 346 nucleotides (nt) upstream of the TG-telomeric repeats, close to the telomere-proximal end of the X-core element present at 1L (Figure 1A and 1C).

We inserted immediately upstream of the start site a cassette containing the *CYC1* 5′ start site and seven tet-operator sequences (TetO7) (Figure 1A and 1C). A mutated tetracycline repressor fusion protein (tTA, tetracycline transactivator), which binds and activates transcription of the transgenic promoter in the absence of doxycycline (Dox) was expressed in this strain from the *ADE2* locus [34]. Addition of Dox to the medium blocks binding of the tTA to the TetO7 sequences thereby preventing transcription. Under promoter-induced conditions in the absence of Dox we mapped the transcription start site of the TetO7-1L transgenic strain as above. One major start site corresponding to the transgenic *CYC1* transcription start site (408 nt upstream of the TG-telomeric repeats) was obtained as expected (Figure 1C and 1D, lanes 2 and 4).

**Figure 2:**
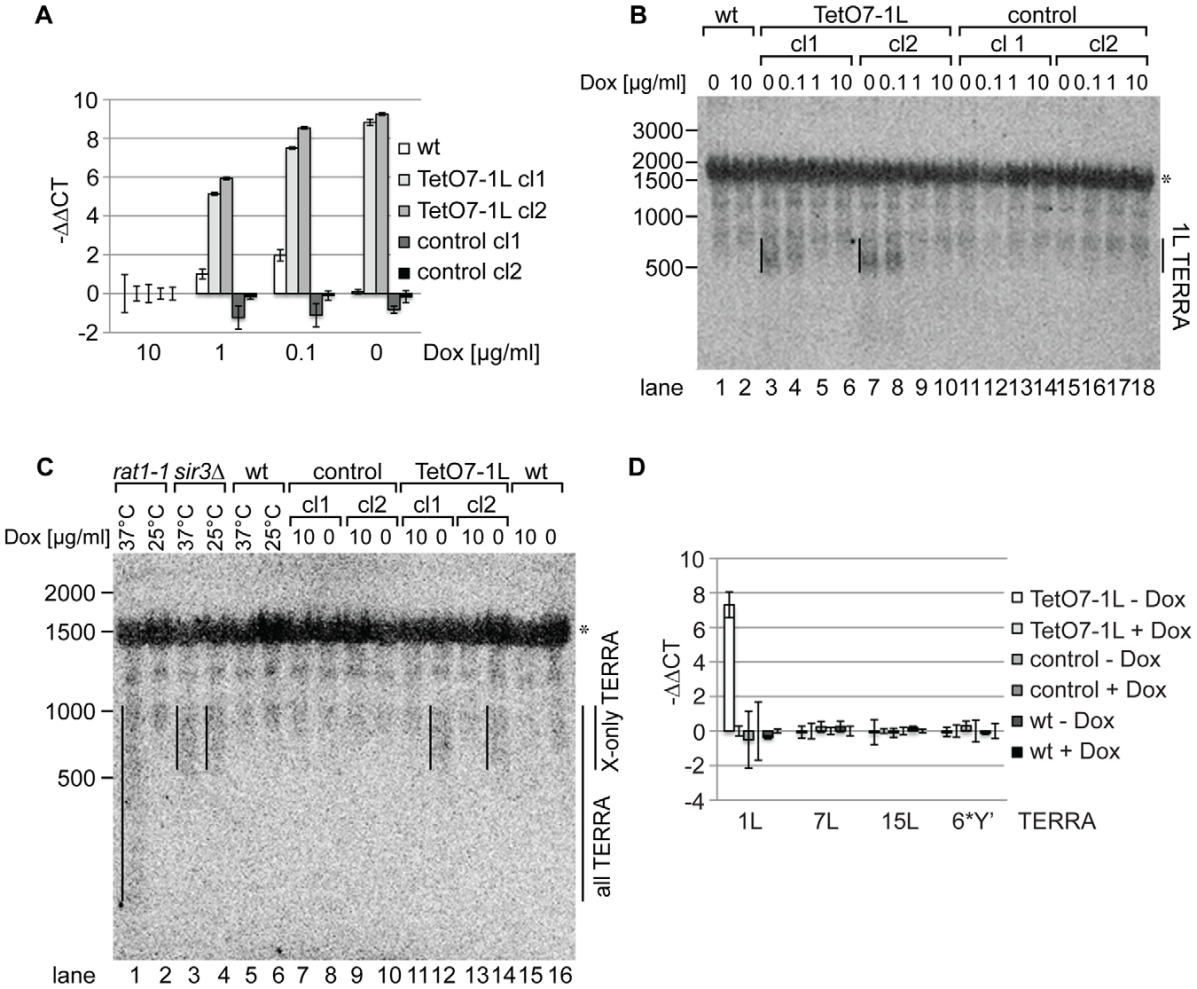
1L TERRA expression depends on the Dox concentration and does not affect other TERRA species. (A) 1L TERRA expression can be modulated by the amount of Dox. qRT-PCR analysis of 1L TERRA in wt, TetO7-1L (two independent clones: cl1 and cl2), and control strains (two independent clones: cl1 and cl2) grown with different Dox concentrations to exponential phase at 30°C in rich medium. −ΔΔCT values of strains grown in 1, 0.1, 0 µg/ml Dox, normalized against actin with standard deviations are shown. The −ΔΔCT values corresponding to each strain grown in 10 µg/ml Dox is arbitrarily set to 0. (for calculations see Materials and Methods published online). (B) Expressed 1L TERRA has a size of 500–800 nt. RNA was extracted from the indicated strains (as in A) grown to exponential phase at 30°C in rich medium. 15 µg of RNA was loaded per lane on a 1.2% formaldehyde/agarose (FA) gel and analyzed by Northern blot analysis. The Northern blot was hybridized with a 5′ end-radiolabeled CA oligonucleotide detecting telomeric GU repeats in 1L TERRA. The 1L TERRA signal is marked with a line. An asterisk marks an unspecific band detected with this probe. RNA marker sizes are indicated at the left in nt. (C) The size of 1L TERRA expressed from TetO7-1L is comparable to the size of TERRA species expressed from other X-only telomeres in a *sir3*Δ mutant. RNA was extracted from the indicated strains grown to exponential phase at 30°C in rich medium. The temperature-sensitive *rat1-1* mutant, wt and *sir3*Δ strains were grown at 25°C to exponential phase in rich medium before the culture was split and either shifted to 37°C or maintained at 25°C for 1 h followed by RNA extraction. 15 µg of RNA was analyzed by Northern blot as described in (B). TERRA transcribed from Y′ and X-only telomeres (all TERRA) are enriched in *rat1-1* cells at non-permissive temperature. *sir3*Δ specifically increases TERRA levels transcribed from X-only telomeres, such as telomere 1L, independently of the temperature [33]. The asterisk marks an unspecific band. RNA marker sizes are given to the left in nt. (D) 1L TERRA expression does not affect the levels of TERRA species transcribed from other telomeres. qRT-PCR analysis of TERRA transcribed from X-only telomeres 1L, 7L, 15L, or from 6 different Y′ telomeres (6*Y′). The indicated strains were grown in rich medium with (+) or without (−) Dox to exponential phase at 30°C. Average −ΔΔCT values of three independent biological replicates normalized against actin with standard deviation are shown. −ΔΔCT values of each strain grown in +Dox is arbitrarily set to 0.

We measured 1L-derived TERRA levels by quantitative reverse transcription PCR (qRT-PCR) in a wild type strain, control strains (two independently generated strains) containing a cassette lacking the TetO7 and *CYC1* sequences (Figure 1A), and TetO7-1L strains (two independently generated strains) containing the TetO7 *CYC1*-cassette in absence and presence of different concentrations of Dox (Figure 2A). In absence of Dox, TERRA 1L was induced >200 fold in the two TetO7-1L transgenic strains. At 1 µg/ml Dox, the induction was around 30 fold and at 10 µg/ml, TERRA 1L was similar in wild type, control and TetO7-1L strains. Under induced conditions (0, 0.1 µg/ml Dox), TERRA 1L was also detectable on Northern blots (Figure 2B, lanes 3, 4, 7, 8). The transcript size ranged between 500 and 800 nt, which we deduce corresponds to a subtelomeric part of 408 nt (Figure 1A, 1C) and a telomeric UG-tract of at least 100 nt. Levels and length of induced 1L TERRA expression are comparable to total TERRA seen in a *sir3Δ* strain (Figure 2C, compare lanes 3 and 4 with 12 and 14; Figure S1). Deletion of *SIR3* induces TERRA mostly from X-only telomeres but not from telomeres containing also Y′ elements [33]. In contrast the *rat1-1* mutation leads to TERRA upregulation from all chromosome ends including Y′ element containing telomeres, giving rise to shorter telomeric transcripts that are also detected on the Northern blot (Figure 2C, lane 1). This is consistent with the position of the transcriptional start site of TERRA transcribed from Y′ element containing telomeres (8L, 8R, 12L-YP1, 12R-YP2, 13L, 15R), which is close to the 3′end of the Y′ ORF as determined by 5′RACE (Figure S2). TERRA transcribed from telomere 8R contains 173 nt of subtelomeric sequence (Figure S2B), which is considerably less than the subtelomeric sequence transcribed at the X-only TERRA 1L (346 nt). We also determined by qRT-PCR whether upregulation of TERRA from TetO7-1L would impact on TERRA levels from other chromosome ends (Figure 2D). 7L and 15L TERRA correspond like 1L to X-only containing telomeres, while 6*Y′ TERRA designates a population of TERRA molecules transcribed from 6 different Y′ element containing telomeres. Full induction of 1L TERRA in absence of Dox did not alter TERRA levels from any other chromosome end tested.

### TERRA transcription induces telomere shortening in cis

**Figure 3:**
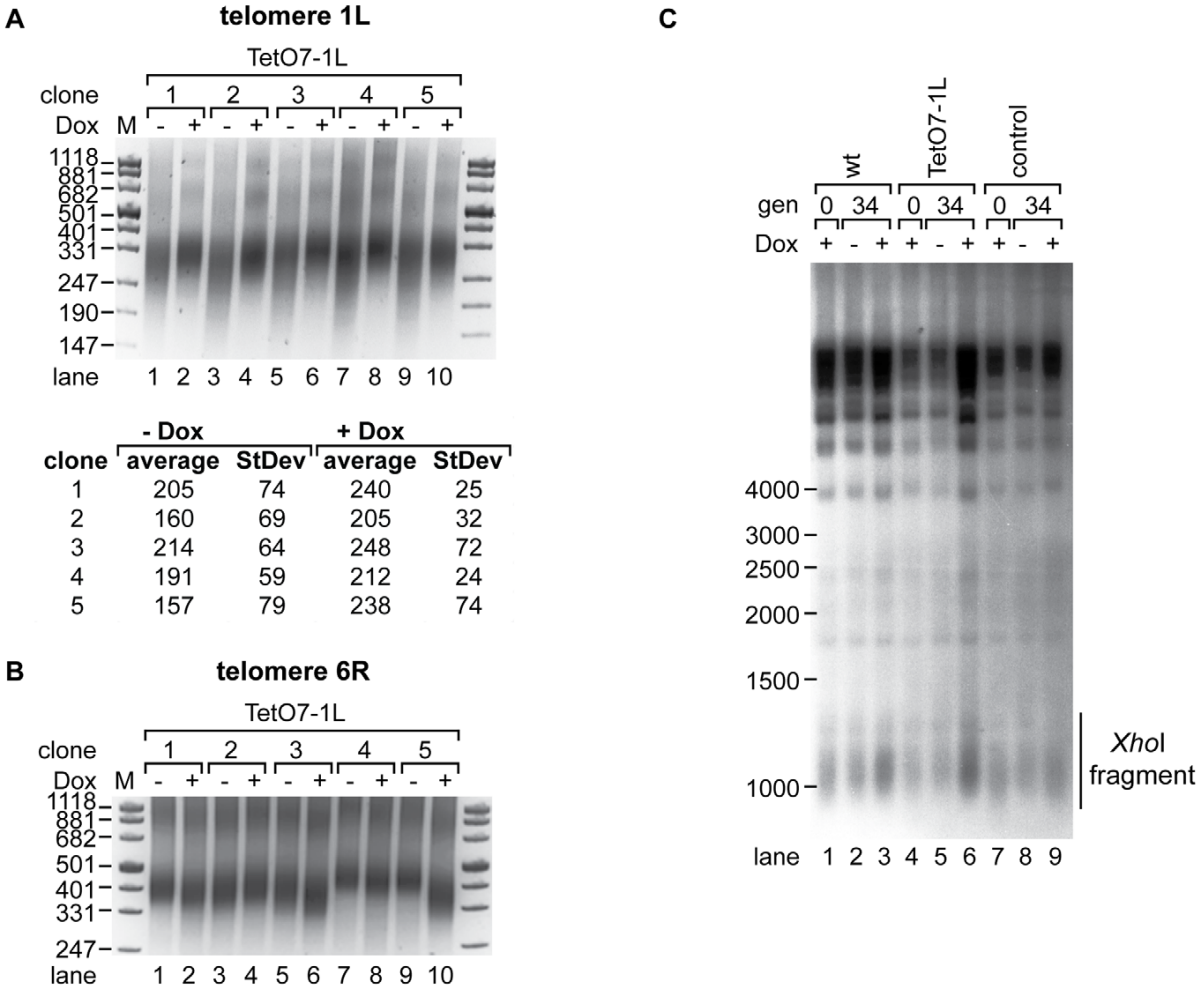
1L TERRA acts in cis and leads to shortening of telomere 1L. (A) 1L TERRA expression leads to shortening of telomere 1L. *Upper panel:* DNA was extracted from five independent clones of the TetO7-1L strain grown at 30°C on YPD plates with (+) or without (−) Dox for 25 generations and analyzed by telomere PCR for telomere 1L on a 2.5% agarose gel. Marker (M) is given in bp. *Lower panel:* 1L telomere PCR products were TOPO cloned and sequenced. Shown is the average length of the TG-tract under inducing (−Dox) and non-inducing (+Dox) conditions with standard deviations. 10 sequences were analyzed for each condition. (B) Telomere 6R is not affected by expression of 1L TERRA. Telomere PCR for telomere 6R performed with DNA from (A). (C) 1L TERRA expression does not affect the average length of Y′ containing telomeres. DNA was extracted from the indicated strains grown as in (A) and digested with *Xho*I before Southern blot analysis. The Southern blot was hybridized with a 5′ end-radiolabeled CA oligonucleotide detecting telomeric TG repeats of yeast telomeres. The telomeric TG repeat signals are highlighted with a line (*Xho*I fragment). Marker (M) is given in bp.

In order to determine effects of TERRA transcription on telomere length, we induced transcription in five independent clones of strain TetO7-1L for 25 generations (see Figure S3A for the quantified TERRA levels of this and Figure S3B–S3H for all subsequent experiments). We measured telomere length at 1L and 6R by telomere PCR [35] (Figure 3A and 3B) and at Y′ element containing telomeres by Southern blotting (Figure 3C). In all five clones, induction of TERRA led to clear telomere shortening at 1L with a concomitant increase in telomere length heterogeneity (Figure 3A upper panel, lanes 1, 3, 5, 7, 9). We sequenced the telomere PCR products from each clone (10 sequences per condition). This revealed that telomere 1L shortened by an average of 40 nucleotides upon 1L TERRA expression (Figure 3A lower panel). The increased length heterogeneity observed on the gel upon 1L TERRA induction was reflected in a higher standard deviation of sequence lengths (Figure 3A). The sequence analysis also confirmed that telomere 1L was specifically amplified by telomere PCR and that the C-tail length varied barely between 18–22 nucleotides. Finally, when performing independent telomere PCR on the same DNA sample, the product lengths were indistinguishable (Figure S4A). These experiments confirm the robustness of the telomere PCR method as here implemented which is in agreement with previous experiments [35]. At telomere 6R, we observed slight telomere length variations in the five different clones (Figure 3B). Telomere length heterogeneity has been described before in different clonal populations of the same strain when analyzing individual chromosome ends (see e.g. [36], [37]). Importantly, however, the length changes at 6R did not correlate with the presence and absence of Dox as was consistently observed for telomere 1L in Figure 3A and subsequent experiments. In order to analyze the lengths of the large population of Y′ element containing telomeres on a Southern blot, we digested genomic DNA with *Xho*I leading to release of terminal telomeric restriction fragments of around 1.3 kb and we hybridized the blot with a telomere-specific oligonucleotide probe (Figure 3C). 1L transcription did not have notable effects on the average telomere lengths of Y′ containing telomeres nor did Dox addition have consistent effects on telomere length of control strains though clonal variations were observed for telomere 6R (Figure S4B, S4C). Altogether, these experiments indicate that telomere transcription promotes telomere shortening *in cis* but not *in trans*.

We tested if transcription-mediated telomere shortening of TetO7-1L was reversible upon transcriptional repression with Dox (Figure S3B). Telomere shortening in the TetO7-1L strain that had been grown under induced conditions for approximately 111 generations was reverted partially but not completely upon addition of Dox for 25 generations (Figure S5, lanes 3 and 4), which can be explained by the still elevated levels of 1L TERRA after Dox addition for 25 generations (Figure S3B). In a reverse experiment, 25 generations of induced transcription was sufficient to induce telomere shortening at telomere 1L (Figure S5, lanes 9 and 10). We conclude that TERRA induced telomere shortening is reversible.

### TERRA accelerates telomere shortening in the absence of telomerase

**Figure 4:**
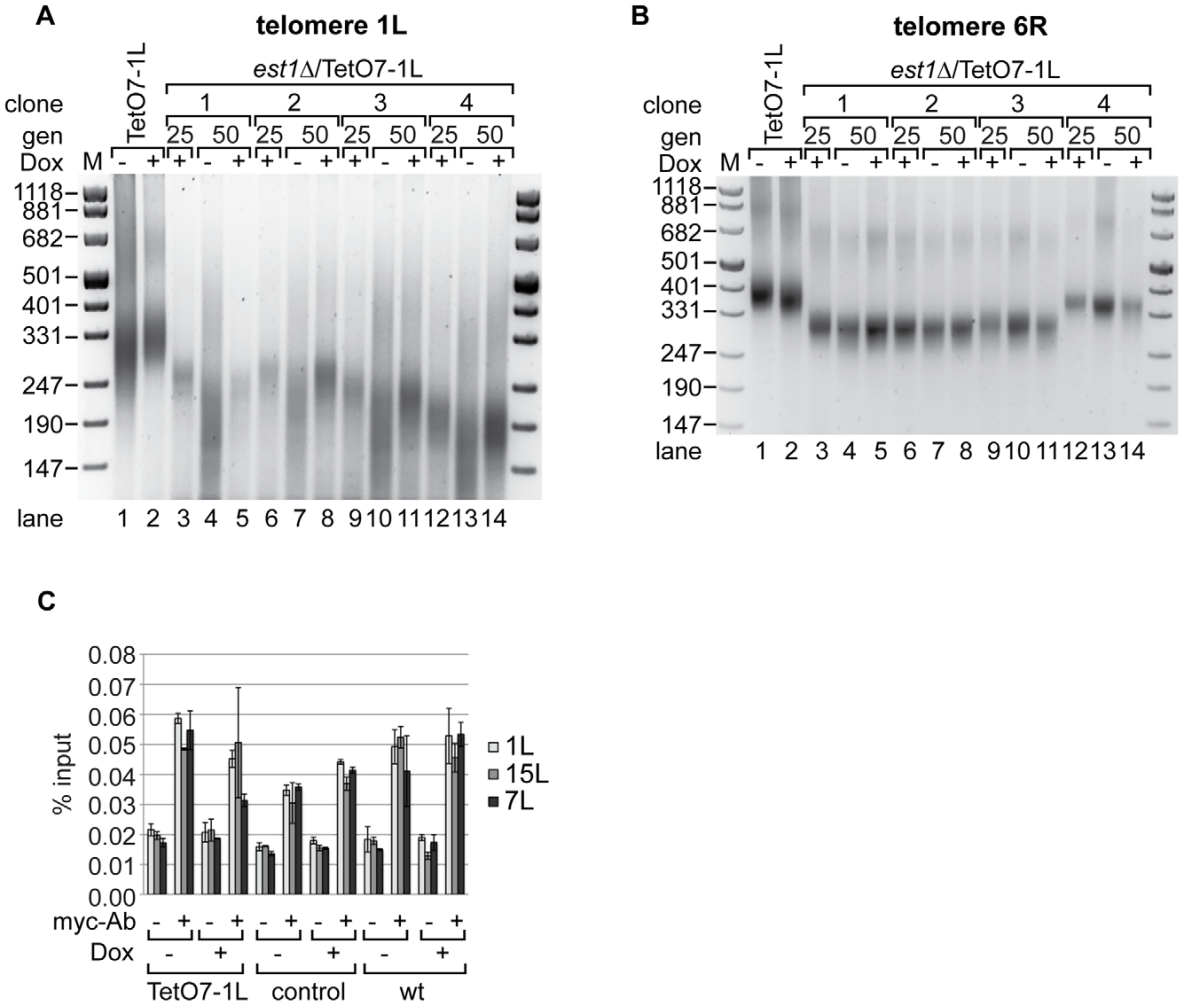
Absence of telomerase increases the shortening of telomere 1L upon 1L TERRA expression. (A) DNA was extracted from TetO7-1L, and *est1*Δ/TetO7-1L (four independent clones) strains grown for 25 generations (gen) at 25°C on YPD plates containing 10 µg/ml Dox (lanes 3, 6, 9, 12), before plating them on YPD plates with (+) (lanes 5, 8, 11, 14) or without (−) Dox (lanes 4, 7, 10, 13) for additional 25 generations. Telomere length at 1L was analyzed by telomere PCR on a 2.5% agarose gel. Marker (M) is given in bp. (B) 1L TERRA expression does not accelerate shortening of telomere 6R in the absence of telomerase. Telomere PCR for telomere 6R performed with DNA from the same strains and growth conditions as in (A). (C) Recruitment of Est2 is not affected by expression of 1L TERRA. Yeast strains were grown for 25 generations at 30°C on YPD plates with (+) or without (−) Dox. Est2-myc associated chromatin was immunoprecipitated after an additional growth to exponential phase in rich medium (−/+Dox) at 30°C (for details see Materials and Methods). The immunoprecipitated telomeres 1L, 7L and 15L were quantified by real-time PCR and expressed as percentage of input. A mock IP lacking the myc-antibody served as negative control. Values of two independent biological replicates with standard deviation are shown.

In order to test effects of TERRA transcription on the telomere shortening rate in the absence of telomerase we deleted the essential telomerase component *EST1* [8] in the TetO7-1L strain. After expanding the *est1Δ* strains for 25 generations under TetO7-repressed conditions in presence of Dox, cells were further propagated in presence and absence of Dox for additional 25 generations. Telomere length was measured at 1L and at 6R with telomere PCR in four independent isolates (Figure 4). As expected, *est1Δ* led to continuous telomere shortening as observed at the 25 and 50 generation time points (Figure 4A). Remarkably, however, upon induction of TERRA 1L (Figure S3C), the telomere shortening rate at 1L was strongly enhanced in the four independently analyzed TetO7-1L *est1Δ* strains (Figure 4A, compare lanes 4 and 5; lanes 7 and 8; lanes 10 and 11; lanes 13 and 14). When the same DNA preparations were analyzed for telomere length at 6R, TERRA induction did not promote telomere shortening (Figure 4B, compare lanes 4 and 5; lanes 7 and 8; lanes 10 and 11; lanes 13 and 14).

We assessed recruitment of Est2 to telomere 1L upon induction of TERRA by chromatin immunoprecipitation (ChIP) [38] (Figure 4C, Figure S3D). For this, the Est2 protein was myc-tagged at its C-terminus downstream of eight glycine residues, which were included as linker. Est2-myc recruitment to telomeres 1L, 7L and 15L were not strongly affected by telomere transcription at 1L (Figure 4C). Expression of TERRA 1L did also not affect overall levels of Est2-myc protein as assessed by Western blotting (Figure S6). Altogether, we conclude that telomere transcription in the absence of telomerase accelerates the telomere shortening rate *in cis*, but telomere transcription *per se* does not strongly affect recruitment of Est2.

### TERRA physically and genetically interacts with the Ku complex

**Figure 5:**
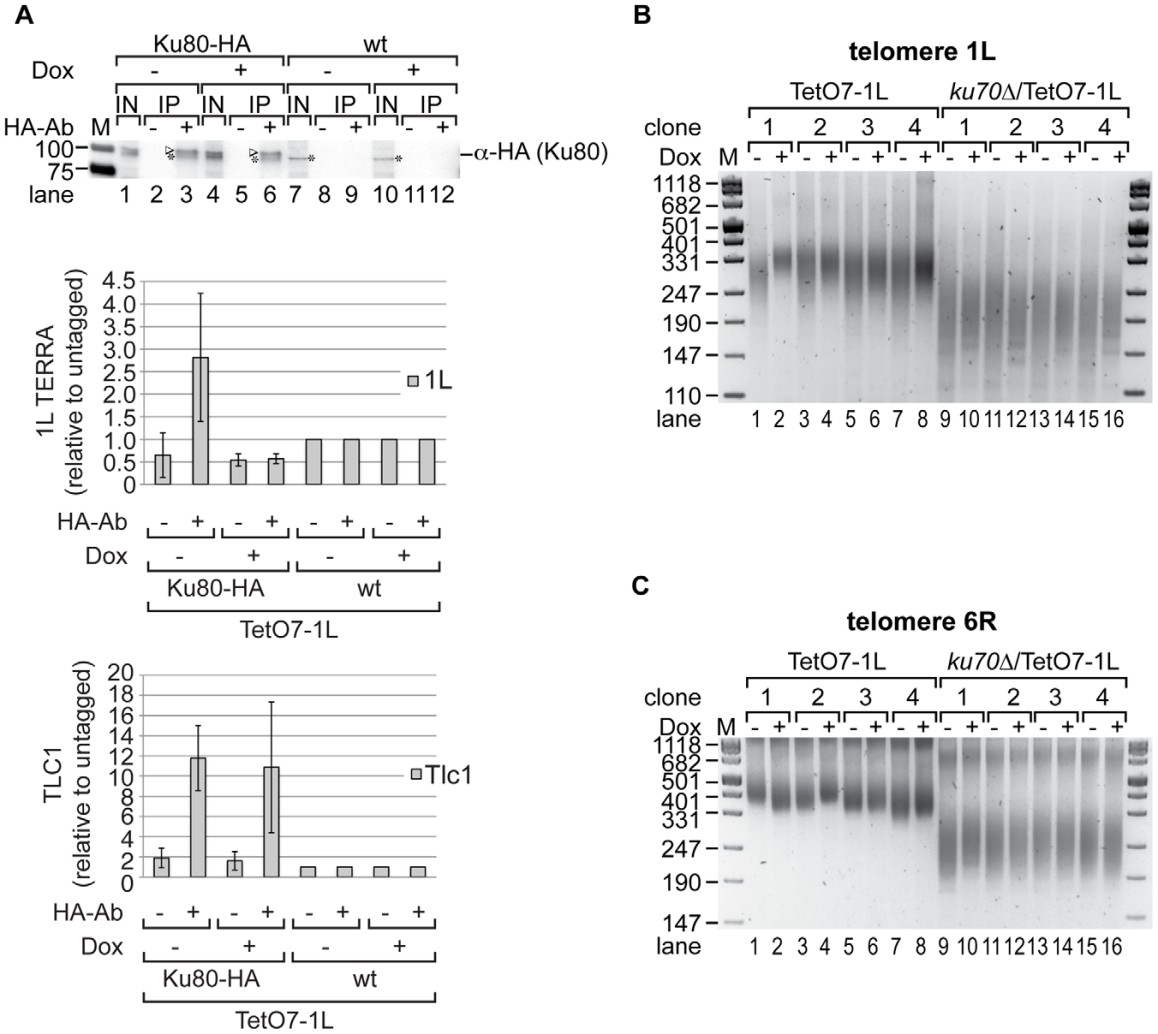
Ku70/80 binds TERRA and *ku70*Δ suppresses the shortening of telomere 1L upon 1L TERRA expression. (A) TERRA is associated with Ku70/80. Yeast strains were grown for 25 generations at 25°C on YPD plates with (+) or without (−) Dox. Ku80-HA associated RNA was immunoprecipitated after an additional growth to exponential phase in rich medium (−/+Dox) at 25°C (for details see Materials and Methods). A mock IP lacking the HA-antibody as well as an untagged strain (wt) served as controls. *Upper panel*: 1L TERRA expression does not influence the specific pull-down efficiency of Ku80-HA in RNA-ChIP experiments as determined by Western blot. After crosslink reversal, input (IN) and immunoprecipitates (IP) were analyzed by PAGE. The Ku80-HA protein band is highlighted by an arrow head; an unspecific band is marked by an asterisk. Marker is given in kDA. *Lower panels*: The immunoprecipitated 1L TERRA and Tlc1 RNA were quantified by RT-PCR and expressed relative to the untagged strains. Values of two independent biological replicates with standard deviation are shown. (B) 1L telomere shortening upon 1L TERRA expression involves Ku70. DNA was extracted from four independent clones of the indicated strains grown at 30°C for 25 generations on YPD plates with (+) or without (−) Dox. Telomere length of telomere 1L was analyzed by telomere PCR on a 2.5% agarose gel. Marker (M) is given in bp. (C) 1L TERRA expression does not affect the length of telomere 6R in the absence of Ku70. Telomere PCR for telomere 6R performed with DNA from (B).

The Ku70/80 dimer binds to telomeres [16] and has also been characterized as RNA binding protein interacting with the telomerase RNA moiety TLC1 in yeast [39], [40]. Therefore, we wondered if Ku70/80 may also bind TERRA. We tagged endogenous Ku80 at its C-terminus with three HA-tags including eight glycine residues as a linker and immunoprecipated Ku80-HA via the tag from crosslinked extracts with similar efficiency in presence or absence of TERRA induction in strain TetO7-1L (Figure 5A, upper panel, compare lanes 3 and 6). An untagged TetO7-1L strain (wt) as well as a mock IP served as negative controls. RNA was purified from crosslinked extracts and co-immunoprecipitates and presence of TERRA, Tlc1 and Act1 RNAs were measured by qRT-PCR (Figure 5A, lower panels). Act1 RNA served as negative control and was not enriched in the HA-tag fraction (Figure S7A). However, 1L TERRA was enriched more than 2.5 fold in the HA-tag fraction over a fraction obtained from the non-tagged, but fully induced TetO7-1L strain (Figure 5A, lower panel, Figure S7B). Pull-down of Tlc1 RNA served as positive control and Tlc1 RNA was enriched more than 10 fold independently of the expression of 1L TERRA (Figure 5A, lower panel). The pull-down efficiency of Tlc1 is similar to a recent publication [40]. Expression levels of Tlc1 RNA were not influenced by induction of 1L TERRA transcription (Figure S7B). We conclude that Ku70/80 binds TERRA even though the affinity for TERRA may be lower than for Tlc1 RNA. Furthermore, since we included a crosslinking step in the experiment, we cannot distinguish direct from indirect physical interactions between Ku70/80 and TERRA. However, our results indicate that TERRA and telomere transcription do not affect the interaction between Ku70/80 and Tlc1 RNA. This is in line with our previous conclusion that TERRA does not interfere with the recruitment of telomerase.

The fact that TERRA can be detected in association with Ku suggested that TERRA may elicit its effects on telomere length *via* this protein. In order to test this possibility, we determined the epistatic relationship of Ku and telomere transcription (Figure 5, Figure S3E). As expected, deletion of *KU70* gave very short telomeres at 1L and 6R (Figure 5B, 5C; Figure S9B, S9C). Importantly, TERRA transcription (25 generations) of 1L in the *ku70*Δ/TetO7-1L strains (four independently generated strains) did not further reduce telomere length of 1L as seen in the *KU70* wt control strains. Nonetheless, a slight increase of telomere length heterogeneity was still observed (Figure 5B, compare telomere length in lanes 9 to 10, 11 to 12, 13 to 14, 15 to 16). On the other hand telomere 1L transcription did not affect the length of telomere 6R as seen before (Figure 5C). Altogether, these results indicate that telomere shortening of TERRA is mediated through Ku.

We tested Ku80 binding to telomere 1L under 1L TERRA inducing and non-inducing conditions by ChIP experiments using HA-tagged Ku80. An untagged but fully induced TetO7-1L strain as well as a mock IP served as negative controls (Figure S7F). Ku80-HA was immunoprecipitated with a similar efficiency independently of 1L TERRA expression (Figure S7D, compare lanes 3 and 6). As shown in Figure S7C, telomere transcription did not significantly alter the binding of Ku70/80 to telomere 1L. In addition, Rap1 binding to telomere 1L was not affected by 1L TERRA expression when analyzed in ChIP experiments (Figure S7E). Binding of Rap1 to the promoter sequence of *CDC19* served as positive control. We conclude that telomere transcription does not interfere with the binding of Ku70/80 and Rap1 to telomeres. Since Rap1 is a binding platform for the Sir complex (Sir2, 3, 4) and the Rif proteins (Rif1, 2), it is unlikely that the binding of these proteins to telomere 1L is affected by 1L TERRA expression. In summary, telomere shortening *in cis* by telomere transcription is due to interference with Ku70/80 function, but it does not phenocopy a Ku deletion, since the shortening phenotype is less severe, and the interaction of Ku and Tlc1 RNA, as well as the recruitment of telomerase were unaffected.

### TERRA–mediated telomere shortening is provoked by Exo1

**Figure 6:**
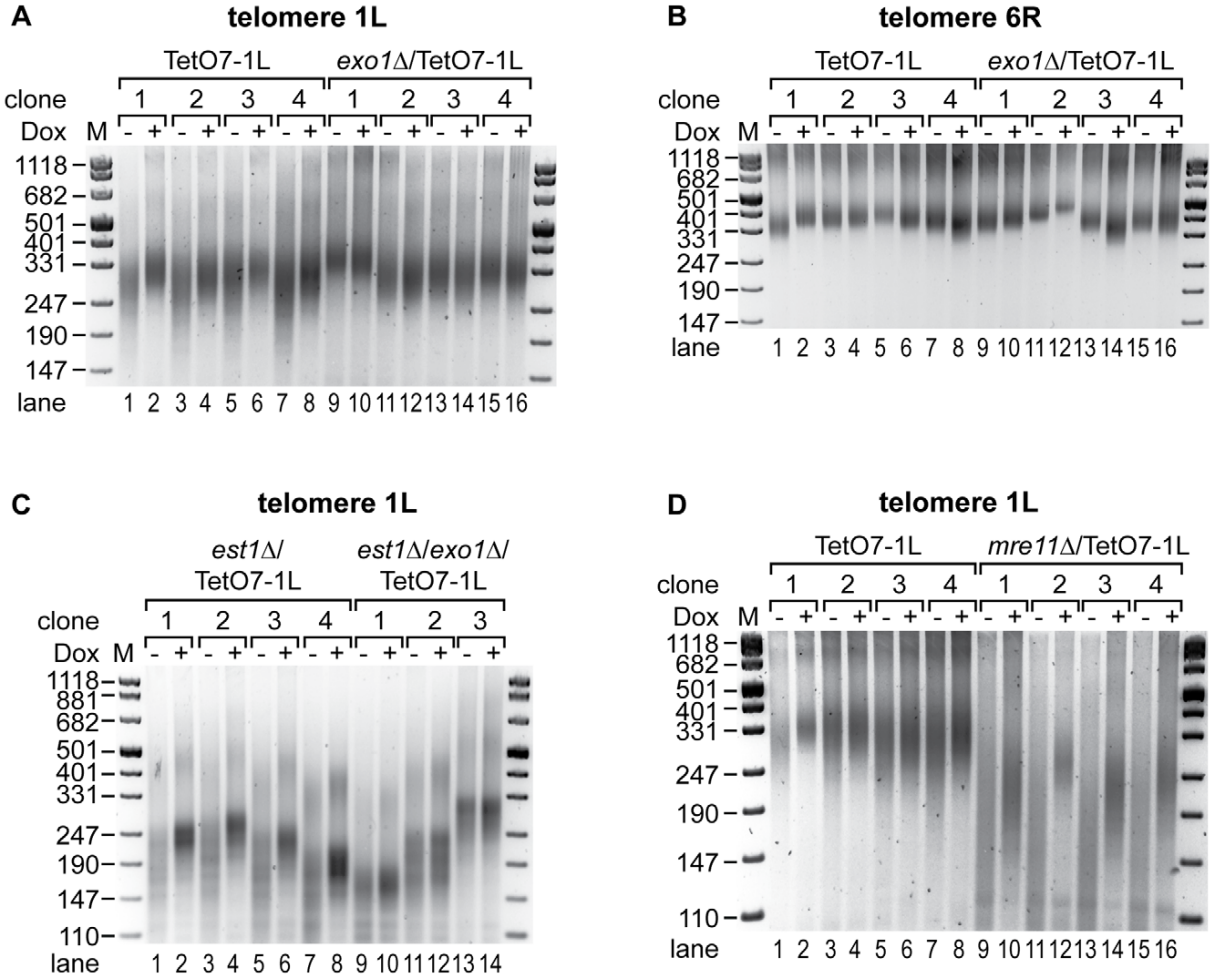
Exo1 mediates shortening of telomere 1L upon 1L TERRA expression, while Mre11 is not involved. (A) TERRA induced shortening of telomere 1L is abolished by deletion of exonuclease 1 (*exo1Δ*). DNA was extracted from four independent clones of the indicated strains grown at 30°C for 25 generations on YPD plates with (+) or without (−) Dox. Telomere length at 1L was analyzed by telomere PCR on a 2.5% agarose gel. Marker (M) is given in bp. (B) Length of telomere 6R is not affected by 1L TERRA expression in the absence of Exo1. Telomere PCR for telomere 6R performed with DNA from (A). (C) Deletion of Exo1 rescues increased telomere shortening of telomere 1L upon TERRA induction in the absence of telomerase. *est1*Δ/TetO7-1L (four independent clones) and *est1*Δ/*exo1*Δ/TetO7-1L (three independent clones) strains were grown for 25 generations at 25°C on YPD plates containing 10 µg/ml Dox, before plating them on YPD plates with (+) (lanes 2, 4, 6, 8, 10, 12, 14) or without (−) (lanes 1, 3, 5, 7, 9, 11, 13) Dox for additional 25 generations. Telomere lengths at 1L were analyzed by telomere PCR on a 2.5% agarose gel. Marker (M) is given in bp. (D) Telomere shortening by 1L TERRA expression is not mediated by Mre11. DNA was extracted from four independent clones of the indicated strains grown at 30°C for 25 generations on YPD plates with (+) or without (−) Dox. Telomere length at 1L was analyzed by telomere PCR on a 2.5% agarose gel. Marker (M) is given in bp.

Telomere shortening is the consequence of incomplete telomere end replication and the action of several nucleases which process chromosome ends in order to generate 3′ overhangs (see Introduction). Notably, Ku70/80 function includes protection of chromosome ends from excessive degradation by the 5′-3′ exonuclease Exo1. In order to test if TERRA transcription promotes nuclease attack at chromosome ends, we determined if deletion of known chromosome end processing activities abolished TERRA-induced telomere shortening. Significantly, deletion of *EXO1* rescued the short telomere phenotype observed at TetO7-1L after TERRA induction (Figure 6A, compare −Dox with +Dox lanes). On the other hand, *exo1Δ* did not notably influence telomere length of 1L in the absence of induced transcription (Figure 6A, compare lanes 2, 4, 6, 8 to lanes 10, 12, 14, 16; Figure S9B, S9C) nor did *exo1Δ* affect length of telomere 6R (Figure 6B). Furthermore, TERRA TetO7-1L expression was not suppressed in *exo1*Δ (Figure S3F), although the overall TERRA levels in the *exo1*Δ strain were slightly decreased in comparison to wt strains (Figure S9A). Moreover, telomere 1L transcription did not induce a global DNA damage response (DDR) in TetO7-1L and the *exo1Δ*/TetO7-1L strains as determined by quantification of Rnr3 mRNA levels (Figure S9A).

To clarify if TERRA induced Exo1 activity mediates accelerated shortening of telomere 1L in the absence of telomerase, *EST1* and *EXO1* were deleted in the TetO7-1L strain. After expanding the *est1*Δ (four independent clones) and *est1*Δ/*exo1*Δ (three independent clones) strains for 25 generations under repressed conditions, cells were propagated for additional 25 generations in presence or absence of Dox. Strikingly, deletion of *EXO1* rescued the TERRA-induced accelerated shortening of telomere 1L in a telomerase deficient strain (Figure 6C, compare −Dox with +Dox lanes in the *est1*Δ and *est1*Δ/*exo1*Δ strains). Expression levels of 1L TERRA were similar in *est1*Δ and *est1*Δ/*exo1*Δ strains (Figure S3G) and length of telomere 6R was independent of 1L TERRA transcription in these strains as expected (Figure S8A). To test if other chromosome end processing activities, like the MRX (Mre11, Rad50, Xrs2) complex, can rescue the telomere shortening phenotype, we deleted *MRE11*. Deletion of *MRE11* caused shorter telomeres as expected (Figure S9B, S9C). Expression of TERRA 1L for 25 generations in the *mre11*Δ strain (Figure S3H) led to further shortening of telomere 1L (Figure 6D, compare lanes 9 to 10, 11 to 12, 13 to 14, 15 to 16), which was not seen at telomere 6R (Figure S8B). Thus, TERRA induced telomere shortening is not dependent on Mre11. We conclude that TERRA promotes Exo1-dependent 5′-3′ resection at chromosome ends *in cis*.

## Discussion

In this paper, we identify the transcription start sites of TERRA and we demonstrate that induction of TERRA at chromosome 1L leads to telomere shortening *in cis*. TERRA transcription was regulated by a Dox-responsive promoter directly upstream of its native transcriptional start site. Since the subtelomeric repeats are crucial elements for telomere function we considered it as important that the induced TERRA transcripts contain all the subtelomeric and telomeric sequences that are also present in native TERRA. Thus our system is different from previous work in which a Gal-promoter was used to induce transcription of telomeric repeats [31]. In our study, we demonstrate that telomere shortening *in cis* is due to increased activity of Exo1 at the transcribed chromosome end as *exo1Δ* rescued the telomere shortening phenotype. Furthermore, we show that the stimulation of Exo1 by telomere transcription occurs in telomerase-positive and telomerase-negative cells.

We also provide evidence that TERRA stimulates Exo1 activity through interfering with Ku function, which represses Exo1 under normal circumstances. First we see in RNA-ChIP experiments that Ku physically interacts with TERRA. Second, we demonstrate that *KU70* deletion abolishes the TERRA-induced telomere shortening phenotype. Thus TERRA requires Ku70 in order to elicit its effects. We therefore suspect that association of TERRA with Ku at the transcribed telomere impairs with Ku's ability to shield telomeres from Exo1. On the other hand, TERRA did not reduce the binding of Ku and Tlc1 RNA nor did it influence in a detectable manner telomerase recruitment to telomeres. Through its interaction with Tlc1 Ku has been implicated in nuclear import and retention of telomerase [40] and its recruitment to telomeres in G1 [38]. Telomeric transcription did also not interfere with Ku70/80 as well as Rap1 binding to telomere 1L as analyzed by ChIP. Thus, telomere transcription does not phenocopy a Ku deletion but only interferes with Ku's function to block Exo1 activity at telomeres.

In previous studies, we demonstrated that human telomerase associates with TERRA, which upon binding acts as a very potent inhibitor of human telomerase [24]. In the current study we do not see signs of telomerase inhibition in yeast upon overexpression of TERRA at telomere 1L. This conclusion is based on the fact that deletion of *EXO1* fully restored 1L TERRA-expression induced telomere shortening in the presence and absence of telomerase. In addition, TERRA expression did not interfere with Tlc1 RNA binding by Ku nor with Est2 recruitment to telomere 1L. Thus yeast telomerase is not regulated by TERRA under the tested conditions. We previously observed with the *rat1-1* mutation effects on telomere lengthening by telomerase but not on the telomere shortening rate [30]. This mutation, however, leads to a stabilization of TERRA transcripts without affecting the rate of TERRA transcription. Thus, the here discovered stimulation of Exo1 may depend on transcription rather than on overall TERRA levels.

Under the optimal growth conditions generally employed in the laboratory, TERRA is scarce and it may not severely impact on Exo1 activity at chromosome ends. However, strongly increased TERRA levels were observed in human ALT cells in which telomeric DNA is maintained by recombination pathways [41] and similarly we observed TERRA upregulation in yeast ALT cells, which survived telomere loss upon telomerase deletion (unpublished results). An increase in overhang lengths of telomeres in ALT cells should facilitate recombination and our results suggest that TERRA might trigger ALT through stimulation of Exo1. As discussed in the Introduction, TERRA is also increased in human ICF syndrome patient derived cells [29]. In patient-derived cell lines telomeres are very short and many chromosome ends lack detectable telomeres altogether. It was suspected that the short telomere phenotype in ICF was mainly due to the inhibition of telomerase by TERRA. Our results linking TERRA to Ku and Exo1 function suggest yet another mechanism. It will be important to determine if Exo1 contributes to the dramatic telomere shortening phenotype seen in these patients and if interference with Exo1 function may reduce the chromosomal instability observed in this disease.

## Materials and Methods

### Yeast strains, construction, growth conditions, and oligonucleotides

All yeast strains used in this study were derived from BY4741 and are listed in Table S1. Growth in YPD medium or on YPD plates supplemented with 10 µg/ml Dox is referred to as +Dox. −Dox refers to growth in Dox-free YPD medium or on YPD plates. Lithium acetate transformation of yeast strains was performed as described [42]. Oligonucleotides used in this study are listed in Table S2.

#### Construction of an inducible TERRA system at telomere 1L

Strain LVPS-5 was generated by replacing *TRP1* (including start and stop codon) with NAT in YNI157 using oligonucleotides oVP22/23 and pAG25 (Euroscarf) as template as described [43]. The tetracycline transactivator (tTA) was amplified from pUG6-tTA (Euroscarf) using oVP8/9 and transformed into LVPS-5 replacing *ADE2* (including start and stop codon), resulting in strain LVPS-11. Deletion of the KAN cassette downstream of the tTA in LVPS-11 by expressing CRE recombinase from pSH47 (Euroscarf) gave strain LVPS-21. LVPS-31/-33 were generated by inserting a *URA3* cassette followed by an *ADH1* terminator, seven tetracycline operator (TetO7) boxes and a cytochrome 1 sequence (*CYC1*) (i.e., PCR product *URA3*_*ADH1*_TetO7_*CYC1*_1L_TERRA amplified with oVP39/oBL1181 from template pLVP010) upstream of the +1 transcription start site of 1L TERRA at telomere 1L of LVPS-21. LVPS-39/-41 were generated by inserting a *URA3* cassette followed by an *ADH1* terminator, lacking promoter sequences (i.e., PCR product *URA3*_*ADH1*_1L_TERRA amplified with oVP39/oBL1181 from template pLVP011) upstream of the +1 transcription start site of 1L TERRA at telomere 1L of LVPS-21.

The Est1 ORF was replaced with a *KAN*MX6 cassette in LVPS-31, -123 using oKF147/oKF148 and template pFA6a-*KAN*MX6 [43]. The Exo1 ORF was replaced with a *HIS3*MX6 cassette in LVPS-21, and -31 using oVP142/oVP146 and template pFA6a-*HIS3*MX6 [43], which resulted in LVPS-118, 123. Deletion of the Mre11 ORF as described above using oVP157/oVP161 resulted in LVPS-131, 132/133. Replacing the Ku70 ORF by a *HIS3*MX6 cassette in LVPS-21, -31 using oVP171/173 gave strains LVPS-137, -138/139. C-terminal tagging of Ku80 with three times HA-tag including eight glycine residues as a linker was performed by amplification of the 3HA-*HIS3*MX6 cassette with oVP187/188 from plasmid pFA6a-3HA-*HIS3*MX6 [43] and transformation of this PCR product into LVPS-31 resulted in strain LVPS-151.

### Plasmid construction

Plasmids used in this study are listed in Table S3. Plasmid pLVP001 was constructed as follows. pGEM3ZF (Stratagene) was cut with *Eco*RI*/Kpn*I and ligated to an accordingly digested PCR fragment containing a *URA3* cassette, which was amplified from pRS306 (Euroscarf) with oVP13/oVP14. A PCR fragment containing the *ADH1* terminator, seven TetO boxes (TetO7) and a cytochrome 1 (*CYC1*) sequence (*ADH1*_TetO7_*CYC1*) was amplified from pCM325 (Euroscarf) using oVP15/oVP16 and cut with *Ava*I*/Bam*HI. The digested PCR product was ligated into *Ava*I*/Bam*HI digested pLVP001, resulting in plasmid pLVP002. A DNA fragment containing the *ADH1* terminator was amplified from pCM325 (Euroscarf) using oVP15/oVP32, cut with *Ava*I*/Bam*HI, and ligated into pLVP001 cut in the same way, giving pLVP009. A PCR product containing a 1L TERRA sequence (ranging from the +1 transcriptional start site of 1L TERRA to 346 bp, immediately upstream of the TG_(1–3)_ repeats) was amplified using oVP37/oVP38 from genomic DNA and was cut with *Bam*HI*/Xba*I. The digested PCR fragment was ligated to *Bam*HI*/Xba*I-digested pLVP002, giving plasmid pLVP010 or to *Bam*HI*/Xba*I digested pLVP009, resulting in plasmid pLVP011.

### Rapid amplification of cDNA ends (5′RACE)

*sir3*Δ (1L TERRA 5′RACE), and wt (Y′ TERRA 5′RACE) cells were grown in 15 ml rich medium at 30°C to an OD_600_ of 0.6 to 0.8. TetO7-1L cells were grown accordingly in YPD medium without (−) or with (+) Dox. 2 ml aliquots were taken at the right OD_600_ and used for RNA isolation followed by three DNase I treatments as described [33]. 5′RACE experiments were carried out using the GeneRacer Kit (Invitrogen). In brief, 5 µg of DNA-free RNA was adjusted to a final volume of 7 µl with H_2_O. 1 µl of 10× CIP buffer, 1 µl of RNaseOut (40 U/µl), and 1 µl of CIP (10 U/µl) were added and incubated at 50°C for 1 h. This treatment dephosphorylates all uncapped RNA species rendering them incompetent for adapter ligation (see below). The treated RNA was precipitated following the instructor's manual, resuspended in 15 µl H_2_O and split into two reactions of 7 µl each. 1 µl of 10× TAP buffer and 1 µl of RNaseOut (40 U/µl) were added to both reactions. TAP treatment was performed in one of the reactions by adding 1 µl TAP (0.5 U/µl). TAP treatment leads to removal of the cap structure leaving a 5′ phosphate group that is required for adaptor ligation. Both reactions were incubated for 1 h at 37°C and precipitated according to the instruction manual and resuspended in 7 µl H_2_O. The decapped RNAs were ligated to 0.25 µg of GeneRacer RNA Oligo as described in the manual. The ligated RNA was resuspended in 13 µl H_2_O. 6 µl of RNA were incubated with 0.4 µl of 25 mM dNTPs, 1 µl of 6 µM oBL1181 (1L TERRA 5′RACE) or of oNI50 (Y′ TERRA 5′RACE) for 5 min at 65°C in a final volume of 13 µl. The reaction was chilled on ice for 1 min, placed at 50°C and 4 µl 5× First Strand Buffer, 1 µl 0.1 M DTT, 1 µl RNaseOut (40 U/µl), 1 µl Superscript III RT (200 U/µl) were added. The reverse transcription conditions were: 50°C for 60 min, 70°C for 15 min. RNase H (2 U) was added, followed by incubation at 37°C for 20 min. Oligonucleotides oBL1181 or oNI50 in combination with GeneRacer 5′primer were used to amplify the 5′end of 1L TERRA by PCR with 2.5 U of Platinum Taq DNA Polymerase (Invitrogen) and 2 µl of cDNA in a 50 µl reaction. Cycling conditions were: 94°C for 2 min, 94°C for 30 sec, 72°C for 1 min, 5 cycles, 94°C for 30 sec, 70°C for 1 min, 5 cycles, 94°C for 30 sec, 62°C for 30 sec, 68°C for 1 min, 20 cycles, and 68°C for 10 min. The nested PCR was performed with 2 µl of the 5′RACE product as template, using oligonucleotides oNI55 and GeneRacer 5′ nested primer with 2.5 U Platinum Taq DNA Polymerase (Invitrogen) in a 50 µl reaction (incubations as follows: 94°C for 2 min, 94°C for 30 sec, 60°C for 30 sec, 68°C for 1 min, 35 cycles, and 68°C for 10 min). The PCR products were separated on a 2% agarose gel. The TAP specific band was cut, eluted, TOPO cloned (Invitrogen) and sequenced.

### RNA isolation, Northern blot analysis, and quantitative real-time PCR (qRT–PCR)

For RNA isolation, cells were grown in rich medium with the indicated concentrations of Dox at 30°C, 220 rpm to an OD_600_ of 0.6 to 0.8. *rat1-1*, *sir3*Δ, and wt cells were grown to an OD_600_ of 0.6 at 25°C, before the culture was split and either maintained at 25°C or shifted to 37°C for 1 h. 2 ml samples were used for RNA isolation, followed by three DNase I treatments as described [33].

For Northern Blot analysis 15 µg of RNA were loaded onto 1.2% formaldehyde agarose (FA) gels, separated by electrophoreses and transferred to Hybond N+ membranes (Amersham) in a tank electroblotter (100 V, 3 h, 4°C) using 0.5× TBE buffer. The RNA was crosslinked to the membrane by UV light. The membranes were pre-hybridized in Church buffer for 1 h at 50°C prior to addition of the ^32^P 5′end-labeled CA oligonucleotide (oBL207). oBL207 was radiolabeled using polynucleotide kinase (PNK). After 12 h of hybridization at 50°C, the membrane was rinsed in 2× SSC containing 0.5% SDS (all SSC buffers were supplemented with 0.5% SDS), followed by two 10 min washes in 2× SSC, one 30 min and one 15 min wash in 0.2× SSC. Signals were visualized on a phosphoimager (Fuji) and band intensities were quantified with AIDA software (Raytest).

Reverse transcription of TERRA was performed as described [33]. In brief, 3 µg RNA was reverse transcribed with 0.5 µM CA oligonucleotide (oBL207; TERRA) and 0.2 µM ACT1 R (oNI212; actin) using 200 U of Superscript III (Invitrogen) in a final volume of 20 µl. Cycling conditions were: 55°C 1 h, 70°C 15 min. Rnr3 was reverse transcribed as above with 0.2 µM RNR3 R (oBL285) and 0.2 µM ACT1 R (oNI212).

qPCR was performed as described [33]. In brief, cDNA was diluted two times with H_2_O. 2 µl of the dilution in a final volume of 20 µl were quantified using Power SYBR Green PCR Master mix (Applied Biosystems) in an Applied Biosystems 7900 HT Fast Real-Time PCR System. Reaction conditions: 95°C for 10 min, 95°C for 15 sec, 60°C for 1 min, 40 cycles. Primer concentrations, sequences and amplified products are listed in Table S2. The specificity of the primers was tested by TOPO cloning [33].

### DNA isolation, terminal transferase-mediated tailing, and telomere PCR

For DNA isolation, single colonies were grown in 5 ml rich medium with the indicated concentrations of Dox at 30°C or 25°C, 220 rpm overnight. DNA of 3 OD units were extracted using the Wizard Genomic DNA purification kit (Promega). Terminal transferase mediated C-tailing was performed as described [35] except that 150 ng of DNA was used. Telomere PCR was performed as described [35] except that 500 µM dNTP, 2.5 U Taq Polymerase (Invitrogen), and 750 nM of oligonucleotides oG18-*BamHI* and oBL1180 (1L telomere PCR) or oJC43 (6R telomere PCR; taken from [12]) were used in a final volume of 40 µl. Cycling conditions for 1L telomere PCR: 94°C for 3 min, 94°C for 30 sec, 61.2°C for 20 sec, 72°C for 20 sec, 35 cycles, 72°C for 5 min. Cycling conditions for 6R telomere PCR: 94°C for 3 min, 94°C for 30 sec, 62°C for 15 sec, 72°C for 20 sec, 44 cycles, 72°C for 5 min. The PCR product of telomere 1L contains 39 bp of subtelomeric sequence, and the PCR product of telomere 6R contains 88 bp of non-TG repeat sequence. The other end of the PCR product harbors 18–22 bp of the tail and a *Bam*HI site, introduced by oligonucleotide oG18-*Bam*HI. The PCR products were analyzed on a 2.5% agarose gel. Where indicated, telomere PCR products were TOPO cloned (Invitrogen) and sequenced using M13rev. For each telomere product 10 different TOPO clones were sequenced.

### Southern blot analysis

Southern blot analysis was performed as described [30]. In brief, 15 µg of DNA were digested with 80 U of *Xho*I overnight at 37°C and loaded on a 1.2% agarose gel. Gels were dried for 1 h at 50°C using a vacuum pump. The DNA was denatured in 20 ml denaturing solution (0.4 M NaOH, 0.6 M NaCl) for 10 min at 50°C and neutralized with 20 ml neutralizing solution (1.5 M NaCl, 0.5 M Trizma-Base, pH 7.5) for 5 min at 50°C. Gels were pre-hybridized in 20 ml Church buffer for 1 h at 50°C prior to addition of the ^32^P 5′end-labeled CA oligonucleotide (oBL207). oBL207 was radiolabeled using PNK. After 12 h of hybridization at 50°C, the gels were rinsed in 0.25× SSC, followed by two 10 min and two 45 min washes in 0.25× SSC. Signals were visualized on a phosphoimager (Fuji) and band intensities were quantified with AIDA software (Raytest).

### Chromatin immunoprecipitation (ChIP)

ChIP was performed as described [30]. In brief, yeast strains were grown in 200 ml rich medium minus or plus Dox at 30°C (Est2-myc ChIP) or 25°C (Ku80-HA, Rap1 ChIP), 220 rpm to an OD_600_ of 0.7–0.8. Cells were crosslinked with 1.2% formaldehyde for 15 min (Est2-myc ChIP) or 25 min (Ku80-HA, Rap1 ChIP) at 25°C, quenched with 360 mM glycine for 5 min at RT, and washed two times with PBS (ice-cold). The pellet was resuspended in 2 ml FA lysis buffer; 700 µl was mixed with 800 µl glass beads, and the cells were lysed using a Fastprep bead breaker (3× 45 sec at level 6.5 M/sec; Thermo Savant). The recovered soluble crosslinked chromatin (13000 rpm, 10 min, 4°C) was resuspended in 2 ml FA lysis buffer containing 0.26% SDS and sonicated for 15 min (Est2-myc ChIP) or 30 min (Ku80-HA, Rap1 ChIP) (30 sec ON, 30 sec OFF; high; Diagenode Bioruptor), yielding fragment sizes of 500 bp to 1 kb (Est2-myc ChIP), or of 150 to 500 bp (Ku80-HA, Rap1 ChIP). The cell debris was removed by centrifugation at 13000 rpm for 15 min at 4°C. For immunoprecipitation of Est2-myc, the chromatin extract (1 mg protein in 1 ml volume) was incubated with 4 µl anti-myc 9B11 mouse antibody (Cell signalling) in the presence of 25 µl (bed volume) Protein A Sepharose (GE Healthcare) overnight at 4°C on a rotating wheel. For immunoprecipitation of Ku80-HA and Rap1, 1.5 mg protein/ml were incubated with 6 µl α-HA 16B12 mouse antibody (Covance) or with 15 µl α-Rap1 (y-300) sc-20167 rabbit antibody (Santa Cruz Biotechnology) and with Protein A beads as mentioned above. A mock IP lacking the antibody or an untagged strain served as controls. 50 µl of the chromatin extract was taken as input sample. Immunoprecipitates were washed, eluted, and the crosslink was reversed as decribed [30]. The crosslink of the input samples was reversed simultaneously. The immunoprecipitated DNA was quantified by real-time PCR using Power SYBR Green PCR Master mix (Applied Biosystems) in an Applied Biosystems 7900 HT Fast Real-Time PCR System and expressed as percentage of input DNA. Primer concentrations, sequences and amplified products are listed in Table S2. 10 µl of the input and 20 µl of the immunoprecipitates were used for Western Blot analysis after reversal of the crosslink.

### RNA–chromatin immunoprecipitation (RNA–ChIP)

Yeast strains were grown in 200 ml YPD with (+) or without (−) Dox to an OD_600_ of 0.7 to 0.8 at 25°C, 220 rpm. Cells were crosslinked with 1.2% formaldehyde for 25 min at 25°C, quenched with 360 mM glycine for 5 min at RT, and washed two times with PBS (ice-cold). The pellet was lysed as described in the ChIP protocol and sonicated for 10 min (30 sec ON, 30 sec OFF; high; Diagenode Bioruptor). The cell debris was removed by centrifugation at 13000 rpm for 15 min at 4°C. For immunoprecipitation of Ku80-HA, the chromatin extract (2 mg protein in 1 ml volume) was incubated with 6 µl α-HA 16B12 mouse antibody (Covance) overnight at 4°C on a rotating wheel. After addition of 25 µl (bed volume) Protein A Sepharose (GE Healthcare) the incubation was prolonged for 2 h 30 min. A mock IP lacking the antibody and a strain lacking the HA-tag at Ku80 served as controls. 40 µl of the extract was taken as input sample. Immunoprecipitates were washed and eluted as described [30]. The crosslink was reversed in the presence of 50 µg RNase-free Proteinase K (Invitrogen) at 65°C for 2 h. RNA (three DNase I treatments) was prepared from 40 µl of the Input samples and 180 µl of the immunoprecipitates and samples were resuspended in 30 µl H_2_O. 1/3 of the RNA was reverse transcribed with 0.5 µM CA oligonucleotide (oBL207; TERRA), 0.2 µM ACT1 R (oNI212; actin), and 0.2 µM TLC1 R (oNI21; TLC1) using 200 U of Superscript III (Invitrogen) in a final volume of 20 µl. Reaction conditions were: 55°C 1 h, 70°C 15 min. The immunoprecipitated RNA was quantified by real-time PCR using Power SYBR Green PCR Master mix (Applied Biosystems) in an Applied Biosystems 7900 HT Fast Real-Time PCR System. RNAs of the tagged strain (LVPS-151) were expressed as fold change over the untagged strain (LVPS-31). Primer concentrations, sequences and amplified products are listed in Table S2. 10 µl of the input and 20 µl of the immunoprecipitates were used for Western Blot analysis after reversal of the crosslink.

### Western blot analysis

Culture samples of 0.5 OD_600_ units (from cultures grown at 30°C, 220 rpm to exponential phase) were taken and centrifuged for 3 min at 3500 rpm. The pellet was resuspended in 1 ml H_2_O. 150 µl of Yex lysis buffer (1.85 M NaOH, 7.5% β-mercaptoethanol) were added and samples were incubated for 10 min on ice. 150 µl of ice-cold 50% TCA (w/v) were added and proteins precipitated on ice for 10 min. The supernatant was removed after centrifugation at 13000 rpm for 5 min at 4°C. The pellets were resuspended in 1× sample loading buffer (0.05 M Tris-HCl pH 6.8, 2% SDS, 10% glycerol, 0.1 M DTT, 0.025% bromphenol blue) to a final concentration of 0.05 OD_600_/µl and boiled for 10 min at 95°C. 0.5 OD_600_ per lane were loaded on a 4–20% SDS-PAGE (precast, Biorad). 2 µl of PageRuler (Thermo Scientific) protein marker were loaded. Gels were blotted for 1 h at 100 V at 4°C in a cable tank blotter onto nitrocellulose membranes (Whatman) in transfer buffer (25 mM Tris base, 190 mM glycine and 20% methanol). Membranes were blocked for 30 min in 5% skim milk in PBST_20_ (1× PBS supplemented with 0.1% Tween 20), followed by incubation with 1/1000 anti-myc monoclonal antibody (9B11, Cell signaling) or with 1/2000 of anti-RNA Pol II monoclonal antibody (8WG16, Abcam), or with 1/1000 anti-HA 16B12 mouse antibody (Covance) overnight at 4°C. After three 15 min washes with PBST_20_, anti-mouse horseradish peroxidase (HRP) coupled antibody (1/3000 in 5% skim milk in PBST_20_, Promega) was incubated for 1 h at RT and washed as above. Western blots were developed using ChemiGlow West (Cell Biosiences) and signals were detected with a FluorChem 8900 (Alpha Innotec).

## Supporting Information

Figure S1 Comparison of 1L TERRA expression in *rat1-1*, *sir3*Δ, and TetO7-1L strains. RNA was extracted from the indicated strains grown to exponential phase at 30°C in rich medium with the given amounts of Dox. The temperature-sensitive *rat1-1* mutant, wt and *sir3*Δ strains were grown at 25°C to exponential phase in rich medium before the culture was split and either shifted to 37°C or maintained at 25°C for 1 h (see Figure 2C). 1L TERRA expression was quantified by qRT-PCR analysis. −ΔΔCT values of strains normalized against actin are shown. The −ΔΔCT values corresponding to each strain grown in presence (+) of Dox or at 25°C are arbitrarily set to 0. TERRA transcribed from Y′ and X-only telomeres (all TERRA) are enriched in *rat1-1* at non-permissive temperature. *sir3*Δ specifically increases TERRA levels transcribed from X-only telomeres, like telomere 1L, independent of the temperature [33]. The amounts of X-only TERRA enriched in *sir3*Δ are comparable to 1L TERRA expression in TetO7-1L.
(TIF)

Figure S2 Characterization of the transcription start site of Y′ TERRA. (A) 5′RACE (absence (−) or presence (+) of Tobacco Acid Pyrophosphatase (TAP)) for mapping the transcription start site (+1) of Y′ TERRA was performed on RNA extracted from wt cells grown to exponential phase in rich medium at 30°C. The RT oligo oNI50 hybridizes to 6 different Y′ telomeres (8L, 8R, 12L-YP1, 12R-YP2, 13L, 15R; see Table S2). Marker (M) is given in bp. (B) Sequence of telomere 8R as an example of a Y′ telomere. The Y′ element sequence (from TG_(1–3)_ repeats to the transcription start site of Y′ TERRA) is highlighted in grey. The 1+ start site of Y′ TERRA is shown in bold, and underlined. Marked are the oligonucleotides used for the RT of the 5′RACE (oNI50) and the ones used in qRT-PCR (oNI62, oNI181) for detection of 6 different Y′ telomeres (6*Y′).
(TIF)

Figure S3 TERRA levels of experiments described in Figure 3, Figure 4, Figure 5, and Figure 6 and Figures S4 and S5. (A) Quantification of 1L TERRA expression with shortening of telomere 1L (see Figure 3, Figure S4). qRT-PCR analysis of TERRA levels in five independent clones of strain TetO7-1L grown at 30°C on YPD plates with (+) or without (−) Dox for 25 generations (gen). 1L and 7L TERRA is transcribed from X-only telomeres. 6*Y′ TERRA corresponds to a population of TERRA transcribed from 6 different Y′ telomeres. −ΔΔCT values of strains normalized against actin with standard deviation are shown. The −ΔΔCT values corresponding to each strain grown in +Dox is arbitrarily set to 0 (for details see Materials and Methods). (B) Quantification of 1L TERRA levels during shut-off or induction of 1L TERRA expression (Figure S5). qRT-PCR analysis of 1L TERRA in wt, TetO7-1L, and control strains grown for 111 generations at 30°C on YPD plates with (+) or without (−) Dox before shutting off or inducing 1L TERRA expression by streaking them for 25 generations on YPD plates with or without Dox. −ΔΔCT values of strains normalized against actin with standard deviation are shown as in (A). (C) Quantification of TERRA levels in the *est1*Δ/TetO7-1L strain grown as described in Figure 4A and B. TERRA levels transcribed from X-only telomeres 1L, 7L, 15L, or from 6 different Y′ telomeres (6*Y′) were analyzed by qRT-PCR analysis. −ΔΔCT values of two independent biological replicates normalized against actin with standard deviation are shown. The −ΔΔCT values are relative to the strain at generation 25 grown in +Dox which is arbitrarily set to 0. (D) Quantification of TERRA in samples used for chromatin immunoprecipitation of Est2-myc (see Figure 4C). Yeast strains were grown for 25 generations at 30°C on YPD plates with (+) or without (−) Dox. RNA was extracted after an additional growth to exponential phase at 30°C in rich medium. TERRA transcribed from X-only telomeres 1L, 7L, 15L, or from 6 different Y′ telomeres (6*Y′) was quantified by qRT-PCR analysis. Average −ΔΔCT values of two independent biological replicates normalized against actin with standard deviation are shown as in (A). (E) Quantification of TERRA expression in a strain deleted for Ku70 (*ku70*Δ) (Figure 5). qRT-PCR analysis of TERRA transcribed from X-only telomeres 1L, 7L, 15L, or from 6 different Y′ telomeres (6′Y) in the *ku70*Δ/TetO7-1L strain grown to exponential phase at 30°C in rich medium as described in Figure 5B. Average −ΔΔCT values of two independent biological replicates normalized against actin with standard deviation are shown as described in (A). (F) Quantification of TERRA expression in a strain deleted for Exo1 (*exo1*Δ) (Figure 6A, 6B). qRT-PCR analysis of TERRA transcribed from X-only telomeres 1L, 7L, 15L, or from 6 different Y′ telomeres (6*Y′) in the *exo1*Δ/TetO7-1L strain grown to exponential phase at 30°C in rich medium as described in Figure 6A. Average −ΔΔCT values of two independent biological replicates normalized against actin with standard deviation are shown as described in (A). (G) Quantification of TERRA levels in the *est1*Δ/TetO7-1L and *est1*Δ/*exo1*Δ/TetO7-1L strains grown as described in Figure 6C and Figure S8A. TERRA levels transcribed from X-only telomeres 1L, 7L, 15L, or from 6 different Y′ telomeres (6*Y′) were analyzed by qRT-PCR analysis. −ΔΔCT values of three independent biological replicates normalized against actin with standard deviations are shown. The −ΔΔCT values corresponding to each strain grown in +Dox is arbitrarily set to 0 (as described in (A)). (H) Quantification of TERRA expression in a strain deleted for Mre11 (*mre11*Δ) (Figure 6D and Figure S8B). qRT-PCR analysis of TERRA transcribed from X-only telomeres 1L, 7L, 15L, or from 6 different Y′ telomeres (6*Y′) in the *mre11*Δ/TetO7-1L strain grown to exponential phase at 30°C in rich medium as described in Figure 6D. Average −ΔΔCT values of two independent biological replicates normalized against actin with standard deviation are shown as described in (A).
(TIF)

Figure S4 Shortening of telomere 1L depends on TERRA expression. (A) The same DNA sample gives reproducible telomere PCR product lengths. DNA was extracted from one clone of the TetO7-1L strain grown at 30°C on YPD plates with (+) or without (−) Dox for 25 generations. Telomere PCR for telomere 1L was performed independently five times with the same DNA samples and analyzed on a 2.5% agarose gel. Marker (M) is given in basepairs (bp). (B) Length of telomere 1L is unaffected in the control strain. DNA was extracted from three independent clones of the indicated strains grown at 30°C with (+) or without (−) Dox for 25 generations and analyzed by telomere PCR for telomere 1L on a 2.5% agarose gel. (C) Telomere 6R is not affected by induced expression of 1L TERRA. Telomere PCR for telomere 6R performed with DNA from (B).
(TIF)

Figure S5 Shortening of telomere 1L can be reversed upon shut-off of 1L TERRA expression. DNA was extracted from wt, TetO7-1L, control strains grown for 111 generations (gen) at 30°C on YPD plates with (+) or without (−) Dox before shutting-off or inducing 1L TERRA expression by streaking them for 25 generations on YPD plates with (+) or without (−) Dox. The DNA was analyzed by telomere PCR for telomere 1L on a 2.5% agarose gel. Marker (M) is given in bp. H_2_O is a water control.
(TIF)

Figure S6 1L TERRA expression does not affect the amount of Est2-myc protein. Yeast strains were grown for 25 generations at 30°C on YPD plates with (+) or without (−) Dox (see Figure 4C). Whole cell protein extract was prepared after an additional growth to exponential phase in rich medium (−/+Dox) at 30°C. Western blot analysis determined the amount of Est2-myc protein. The Rpb1 subunit of RNA polymerase II (RNA Pol II) was used as loading control.
(TIF)

Figure S7 1L TERRA is specifically pulled down with Ku80-HA and 1L TERRA expression does not interfere with binding of Ku70/80 and Rap1 to telomere 1L. (A) Act1 is not enriched in the HA-tagged fraction in RNA-ChIP experiments using Ku80-HA (see Figure 5A). Yeast strains were grown for 25 generations at 25°C on YPD plates with (+) or without (−) Dox. Ku80-HA associated RNA was immunoprecipitated after an additional growth to exponential phase in rich medium (−/+Dox) at 25°C. A mock IP lacking the HA-antibody as well as an untagged strain (wt) served as controls. The immunoprecipitated Act1 RNA was quantified by real-time PCR and expressed relative to the untagged strain. Values of two independent biological replicates with standard deviation are shown. (B) Quantification of TERRA in samples used for RNA-ChIP of Ku80-HA (see Figure 5A). Yeast strains were grown and RNA was extracted as described in A. Transcribed 1L TERRA and Tlc1 RNA were quantified by qRT-PCR analysis. −ΔΔCT values of strains normalized against actin with standard deviation are shown (two independent biological replicates). The −ΔΔCT values corresponding to each strain grown in +Dox is arbitrarily set to 0. (C) Binding of Ku80 to telomere 1L is not affected by expression of 1L TERRA. Yeast strains were grown for 25 generations at 25°C on YPD plates with (+) or without (−) Dox. Ku80-HA associated chromatin was immunoprecipitated after an additional growth to exponential phase in rich medium (−/+Dox) at 25°C (for details see Materials and Methods). The immunoprecipitated DNA for telomere 1L, 7L, and 15L was quantified by real-time PCR and expressed as percentage of input normalized to each strain under +Dox conditions. A mock IP lacking the HA-antibody as well as an untagged strain served as controls. Values of two independent biological replicates with standard deviation are shown. (D) 1L TERRA expression does not influence the pull-down efficiency of Ku80-HA in ChIP experiments (see C) as determined by Western blot. After crosslink reversal, input (IN) and immunoprecipitates (IP) were loaded on a 4–20% SDS-Page. Marker (M) is given in kDA. (E) Binding of Rap1 to telomere 1L is not affected by expression of 1L TERRA. ChIP experiments were performed and analyzed as described in C. A mock IP lacking the Rap1-antibody served as control. Values of two independent biological replicates with standard deviations are shown. (F) Quantification of TERRA in samples used for ChIP of Ku80-HA and Rap1 (see C, E). Yeast strains were grown and RNA was extracted as described in A. TERRA levels transcribed from X-only telomeres 1L, 7L, 15L were analyzed by qRT-PCR analysis. Average −ΔΔCT values of two independent biological replicates normalized against actin with standard deviation are shown. The −ΔΔCT values corresponding to each strain grown in +Dox is arbitrarily set to 0.
(TIF)

Figure S8 Expression of 1L TERRA does not affect length of telomere 6R in the absence of Exo1, telomerase and Mre11. (A) Length of telomere 6R is not affected upon TERRA expression in the absence of Exo1 and telomerase. *est1*Δ/TetO7-1L (four independent clones) and *est1*Δ/*exo1*Δ/TetO7-1L (three independent clones) strains were grown as described in Figure 6C. Telomere length of 6R was analyzed by telomere PCR on a 2.5% agarose gel. Marker (M) is given in bp. (B) Length of telomere 6R is not affected upon 1L TERRA expression in the absence of Mre11. DNA was extracted from four independent clones of the indicated strains grown as described in Figure 6D. Telomere length at 6R was analyzed by telomere PCR on a 2.5% agarose gel. Marker (M) is given in bp.
(TIF)

Figure S9 Comparison of TERRA levels in wt, *exo1*Δ, *ku70*Δ, *mre11*Δ. (A) Deletion of Exo1 decreases and deletion of Ku70 increases TERRA levels independent of a DNA damage response (DDR). Deletion of Mre11 (*mre11*Δ induces a DDR, but has no effect on TERRA levels. qRT-PCR analysis of Rnr3 (checkpoint marker for DDR) and TERRA transcribed from X-only telomeres (1L, 7L, 15L) and from 6 different Y′ telomeres (6*Y′) in wt, *exo1*Δ, *ku70*Δ, *mre11*Δ strains. RNA was extracted from these strains grown in rich medium at 30°C to an OD_600_ of 0.7. Average −ΔΔCT values of two independent biological replicates normalized against actin with standard deviation are shown. −ΔΔCT values of the wt strain are arbitrarily set to 0. (B) Deletion of *KU70* and *MRE11* reduces the length of telomere 1L. DNA was extracted from strains grown as described in (A) and analyzed by telomere PCR for telomere 1L on a 2.5% agarose gel. Marker (M) is given in bp. (C) Deletion of *KU70* and *MRE11* reduces the length of telomere 6R as determined by telomere PCR for 6R. DNA was extracted from strains grown as described in (A).
(TIF)

Table S1 Strains used in this study.
(PDF)

Table S2 Oligonucleotides used in this study.
(PDF)

Table S3 Plasmids used in this study.
(PDF)
