# Supplementary figures and images for: TERRA Promotes Telomere Shortening through Exonuclease 1–Mediated Resection of Chromosome Ends
Source: PLoS Genet. 2012 Jun 14;8(6):e1002747. doi: 10.1371/journal.pgen.1002747 (PMC3375253; doi:10.1371/journal.pgen.1002747)

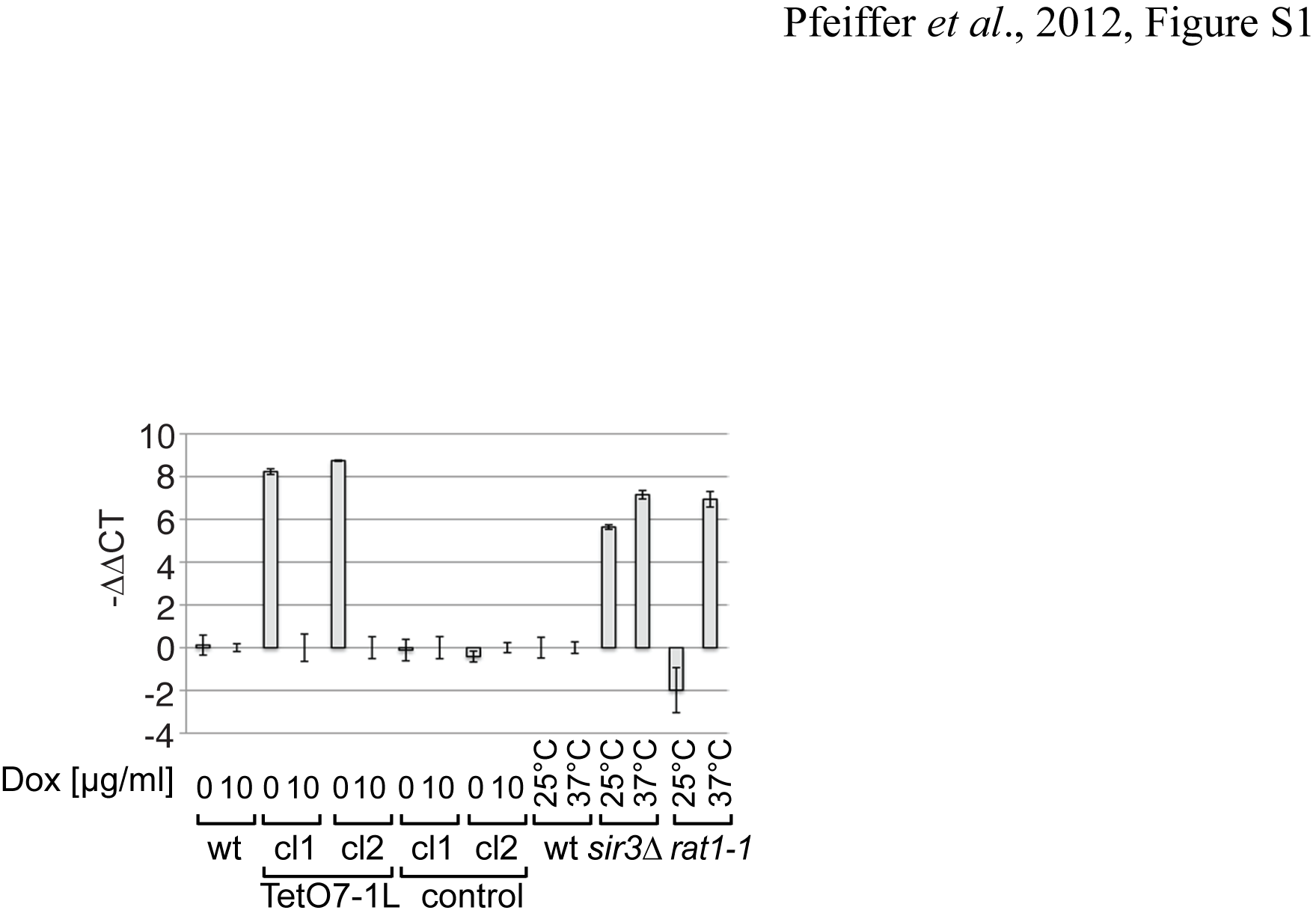

Supplement: Figure S1 — Comparison of 1L TERRA expression in rat1-1, sir3Δ, and TetO7-1L strains. RNA was extracted from the indicated strains grown to exponential phase at 30°C in rich medium with the given amounts of Dox. The temperature-sensitive rat1-1 mutant, wt and sir3Δ strains were grown at 25°C to exponential phase in rich medium before the culture was split and either shifted to 37°C or maintained at 25°C for 1 h (see Figure 2C). 1L TERRA expression was quantified by qRT-PCR analysis. −ΔΔCT values of strains normalized against actin are shown. The −ΔΔCT values corresponding to each strain grown in presence (+) of Dox or at 25°C are arbitrarily set to 0. TERRA transcribed from Y′ and X-only telomeres (all TERRA) are enriched in rat1-1 at non-permissive temperature. sir3Δ specifically increases TERRA levels transcribed from X-only telomeres, like telomere 1L, independent of the temperature [33]. The amounts of X-only TERRA enriched in sir3Δ are comparable to 1L TERRA expression in TetO7-1L. (TIF) [file pgen.1002747.s001.tif]

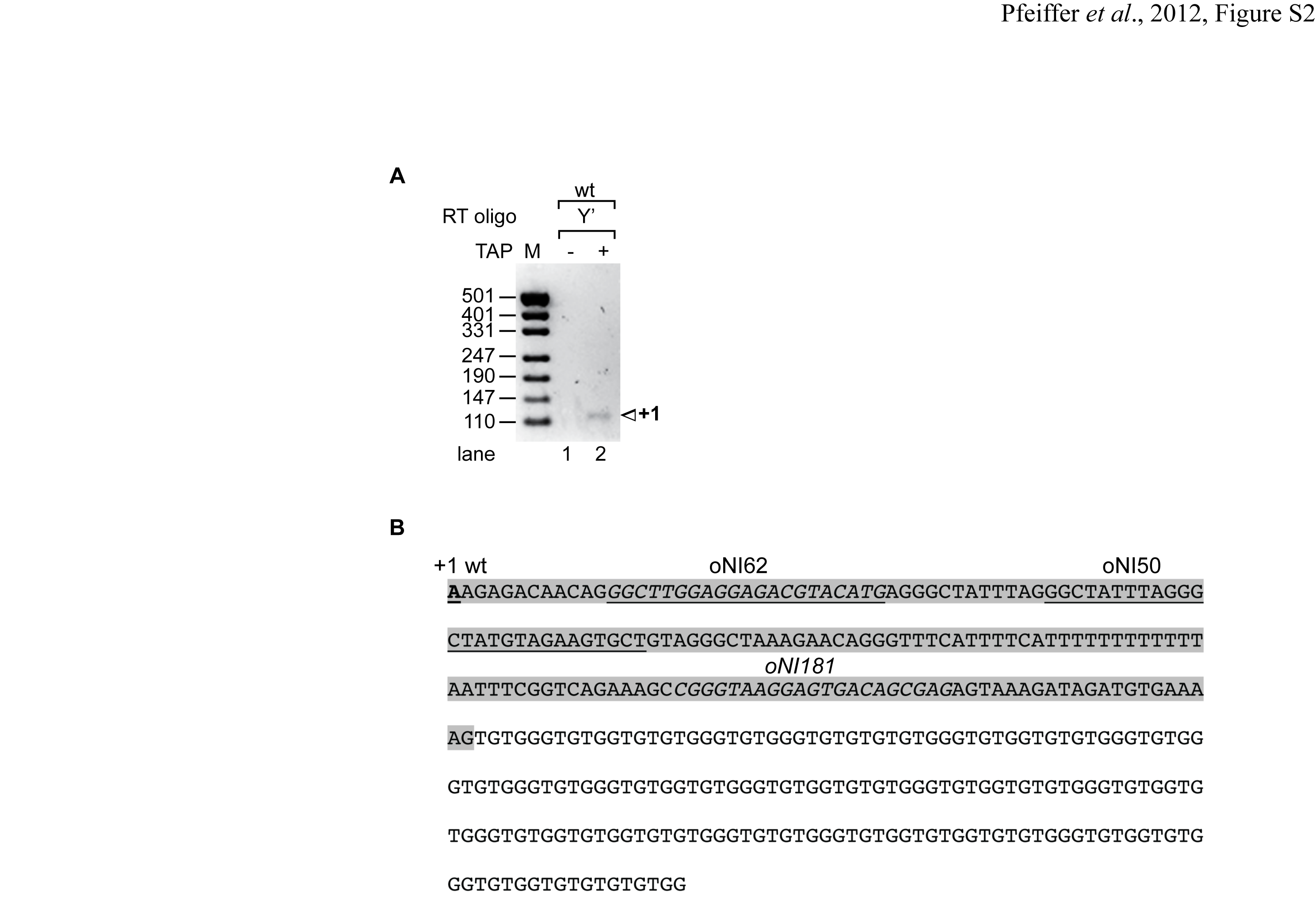

Supplement: Figure S2 — Characterization of the transcription start site of Y′ TERRA. (A) 5′RACE (absence (−) or presence (+) of Tobacco Acid Pyrophosphatase (TAP)) for mapping the transcription start site (+1) of Y′ TERRA was performed on RNA extracted from wt cells grown to exponential phase in rich medium at 30°C. The RT oligo oNI50 hybridizes to 6 different Y′ telomeres (8L, 8R, 12L-YP1, 12R-YP2, 13L, 15R; see Table S2). Marker (M) is given in bp. (B) Sequence of telomere 8R as an example of a Y′ telomere. The Y′ element sequence (from TG(1–3) repeats to the transcription start site of Y′ TERRA) is highlighted in grey. The 1+ start site of Y′ TERRA is shown in bold, and underlined. Marked are the oligonucleotides used for the RT of the 5′RACE (oNI50) and the ones used in qRT-PCR (oNI62, oNI181) for detection of 6 different Y′ telomeres (6*Y′). (TIF) [file pgen.1002747.s002.tif]

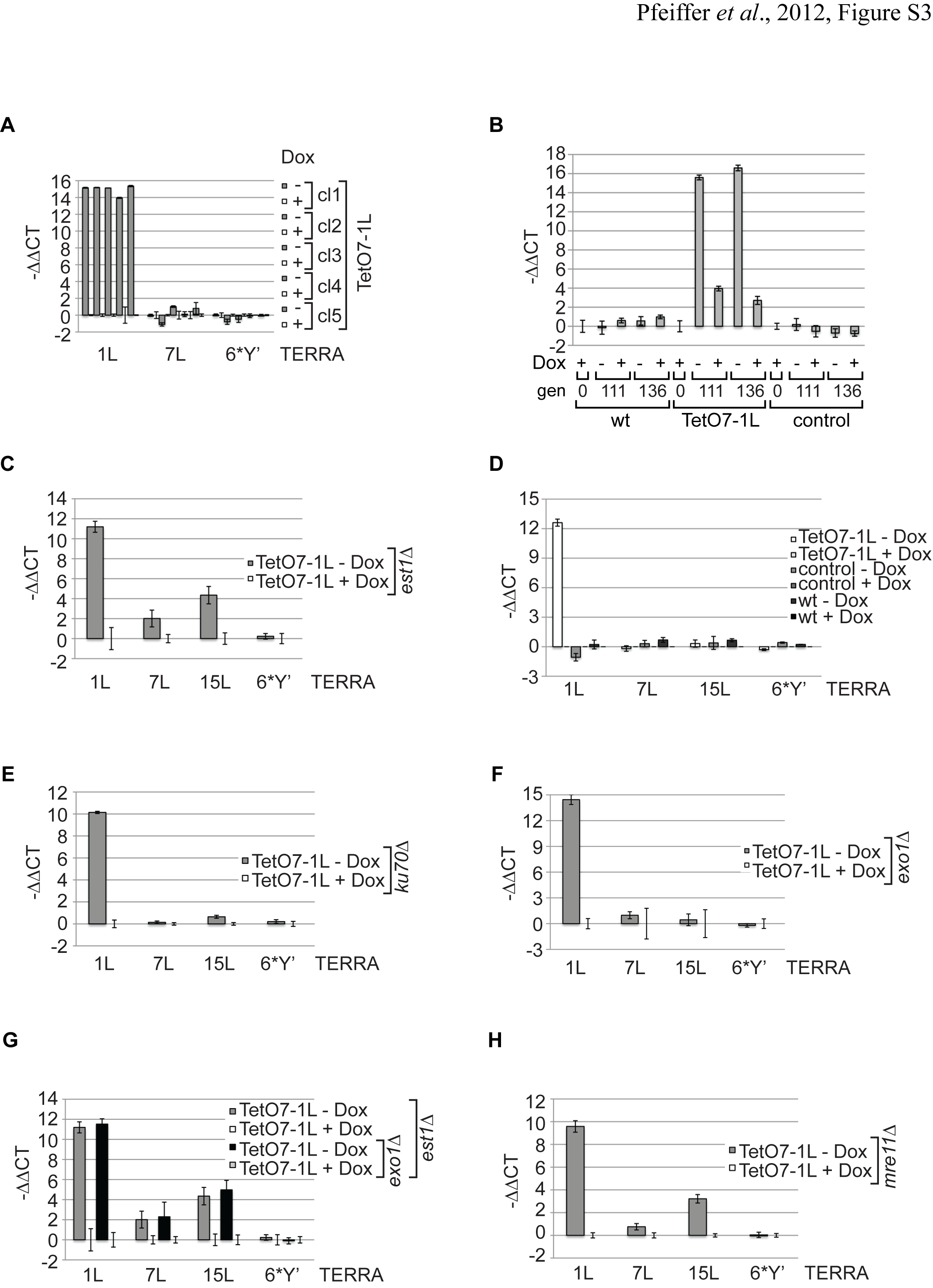

Supplement: Figure S3 — TERRA levels of experiments described in Figure 3, Figure 4, Figure 5, and Figure 6 and Figures S4 and S5. (A) Quantification of 1L TERRA expression with shortening of telomere 1L (see Figure 3, Figure S4). qRT-PCR analysis of TERRA levels in five independent clones of strain TetO7-1L grown at 30°C on YPD plates with (+) or without (−) Dox for 25 generations (gen). 1L and 7L TERRA is transcribed from X-only telomeres. 6*Y′ TERRA corresponds to a population of TERRA transcribed from 6 different Y′ telomeres. −ΔΔCT values of strains normalized against actin with standard deviation are shown. The −ΔΔCT values corresponding to each strain grown in +Dox is arbitrarily set to 0 (for details see Materials and Methods). (B) Quantification of 1L TERRA levels during shut-off or induction of 1L TERRA expression (Figure S5). qRT-PCR analysis of 1L TERRA in wt, TetO7-1L, and control strains grown for 111 generations at 30°C on YPD plates with (+) or without (−) Dox before shutting off or inducing 1L TERRA expression by streaking them for 25 generations on YPD plates with or without Dox. −ΔΔCT values of strains normalized against actin with standard deviation are shown as in (A). (C) Quantification of TERRA levels in the est1Δ/TetO7-1L strain grown as described in Figure 4A and B. TERRA levels transcribed from X-only telomeres 1L, 7L, 15L, or from 6 different Y′ telomeres (6*Y′) were analyzed by qRT-PCR analysis. −ΔΔCT values of two independent biological replicates normalized against actin with standard deviation are shown. The −ΔΔCT values are relative to the strain at generation 25 grown in +Dox which is arbitrarily set to 0. (D) Quantification of TERRA in samples used for chromatin immunoprecipitation of Est2-myc (see Figure 4C). Yeast strains were grown for 25 generations at 30°C on YPD plates with (+) or without (−) Dox. RNA was extracted after an additional growth to exponential phase at 30°C in rich medium. TERRA transcribed from X-only telomeres 1L, 7L, 15L, or from 6 di [file pgen.1002747.s003.tif]

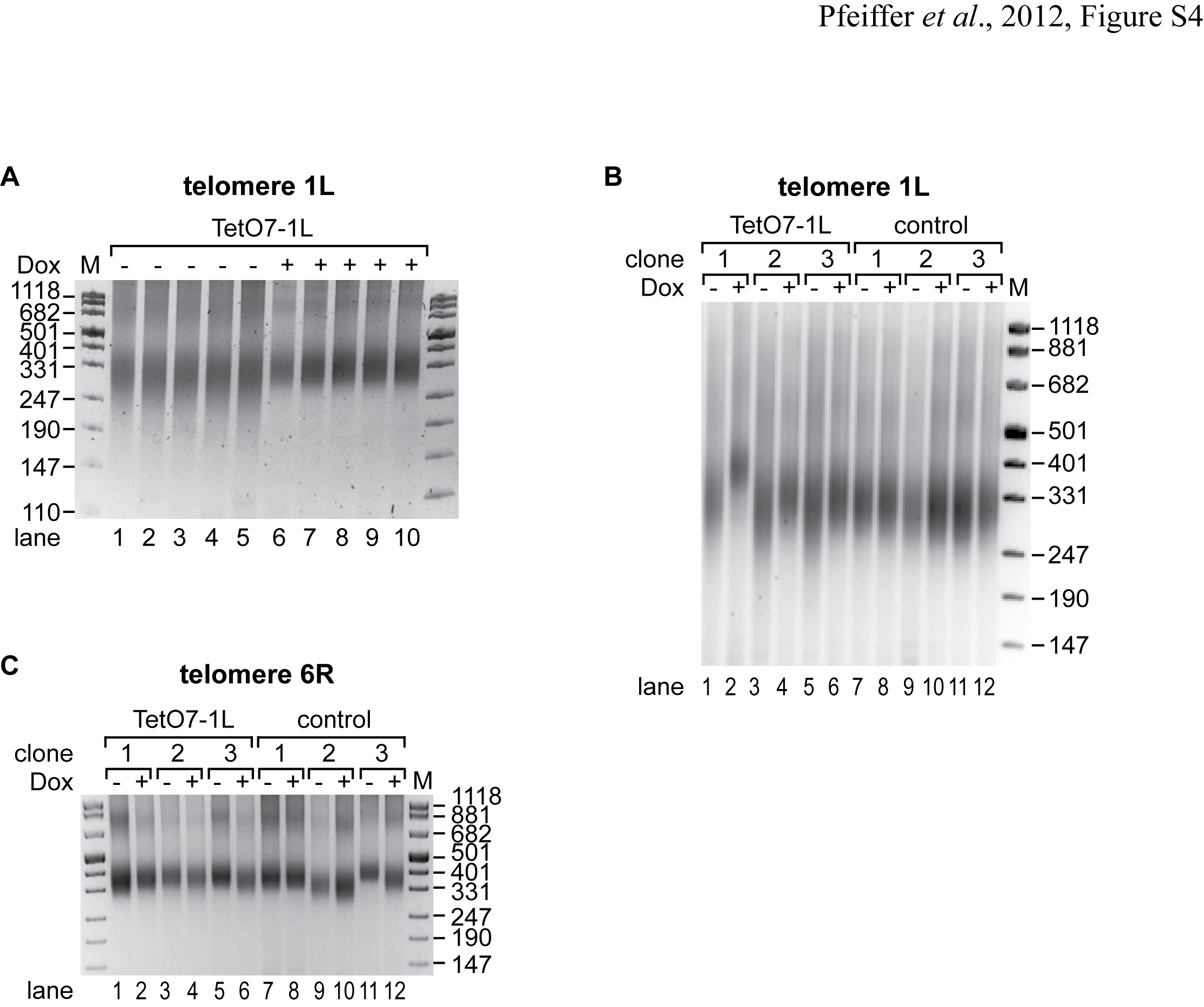

Supplement: Figure S4 — Shortening of telomere 1L depends on TERRA expression. (A) The same DNA sample gives reproducible telomere PCR product lengths. DNA was extracted from one clone of the TetO7-1L strain grown at 30°C on YPD plates with (+) or without (−) Dox for 25 generations. Telomere PCR for telomere 1L was performed independently five times with the same DNA samples and analyzed on a 2.5% agarose gel. Marker (M) is given in basepairs (bp). (B) Length of telomere 1L is unaffected in the control strain. DNA was extracted from three independent clones of the indicated strains grown at 30°C with (+) or without (−) Dox for 25 generations and analyzed by telomere PCR for telomere 1L on a 2.5% agarose gel. (C) Telomere 6R is not affected by induced expression of 1L TERRA. Telomere PCR for telomere 6R performed with DNA from (B). (TIF) [file pgen.1002747.s004.tif]

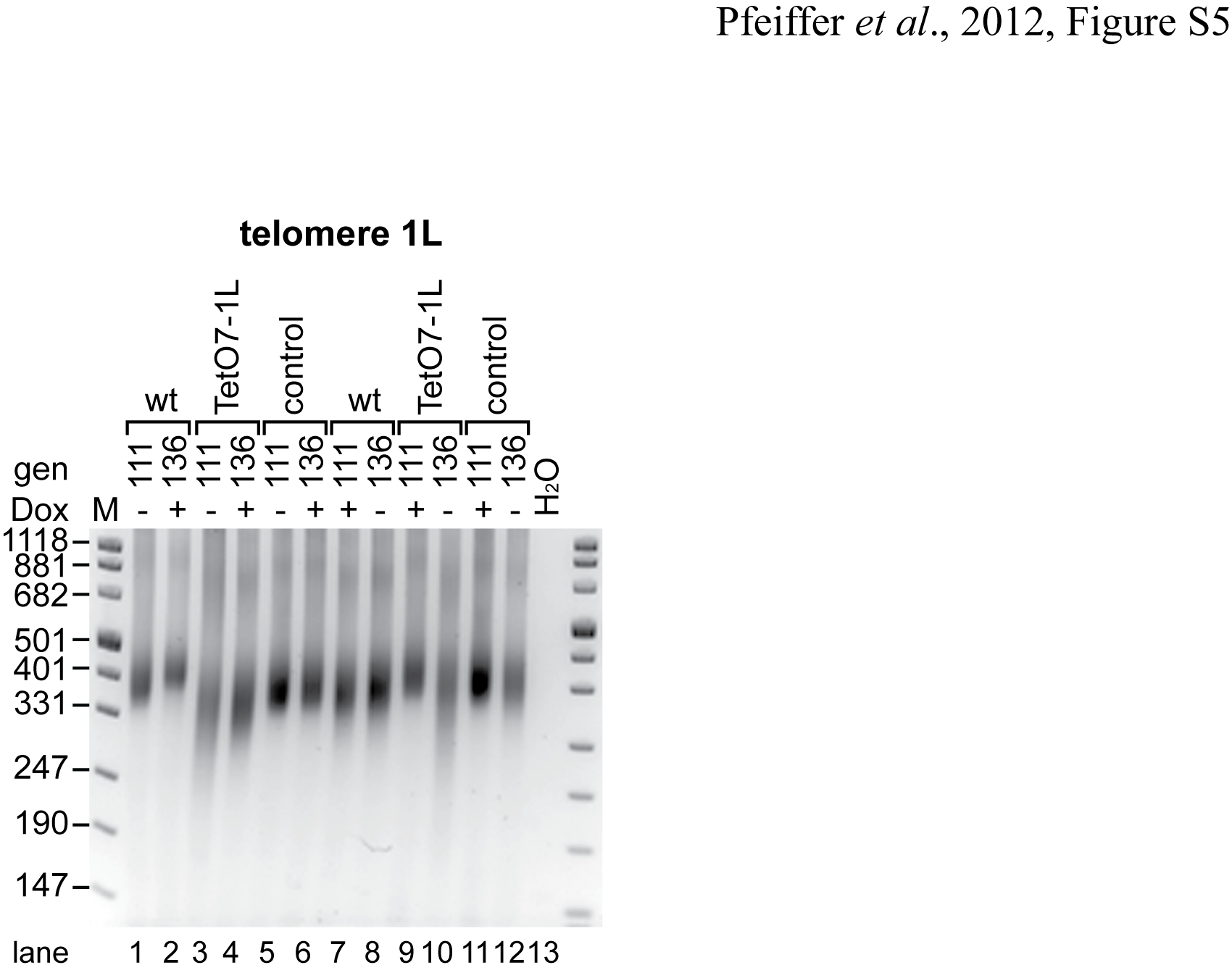

Supplement: Figure S5 — Shortening of telomere 1L can be reversed upon shut-off of 1L TERRA expression. DNA was extracted from wt, TetO7-1L, control strains grown for 111 generations (gen) at 30°C on YPD plates with (+) or without (−) Dox before shutting-off or inducing 1L TERRA expression by streaking them for 25 generations on YPD plates with (+) or without (−) Dox. The DNA was analyzed by telomere PCR for telomere 1L on a 2.5% agarose gel. Marker (M) is given in bp. H2O is a water control. (TIF) [file pgen.1002747.s005.tif]

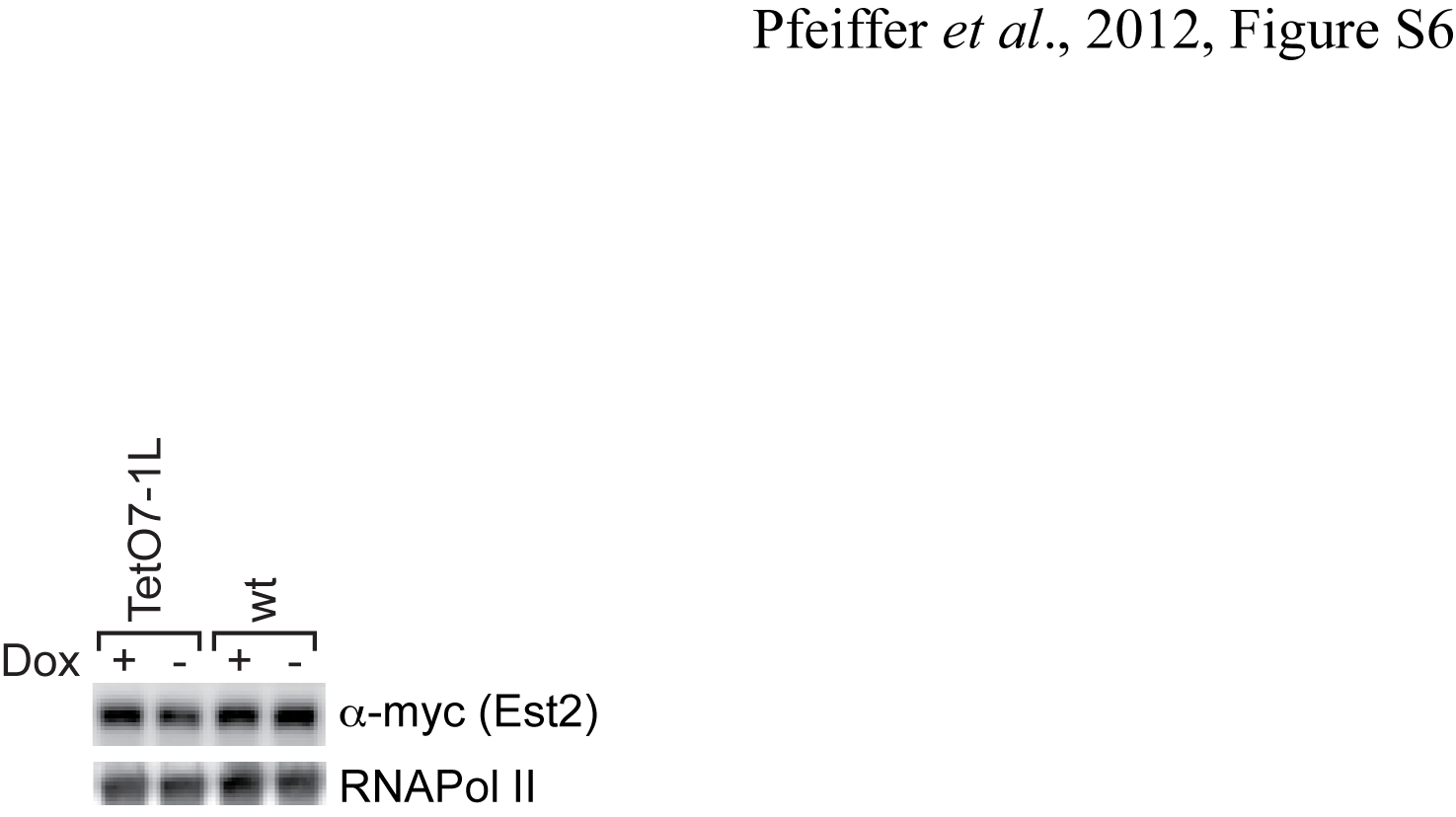

Supplement: Figure S6 — 1L TERRA expression does not affect the amount of Est2-myc protein. Yeast strains were grown for 25 generations at 30°C on YPD plates with (+) or without (−) Dox (see Figure 4C). Whole cell protein extract was prepared after an additional growth to exponential phase in rich medium (−/+Dox) at 30°C. Western blot analysis determined the amount of Est2-myc protein. The Rpb1 subunit of RNA polymerase II (RNA Pol II) was used as loading control. (TIF) [file pgen.1002747.s006.tif]

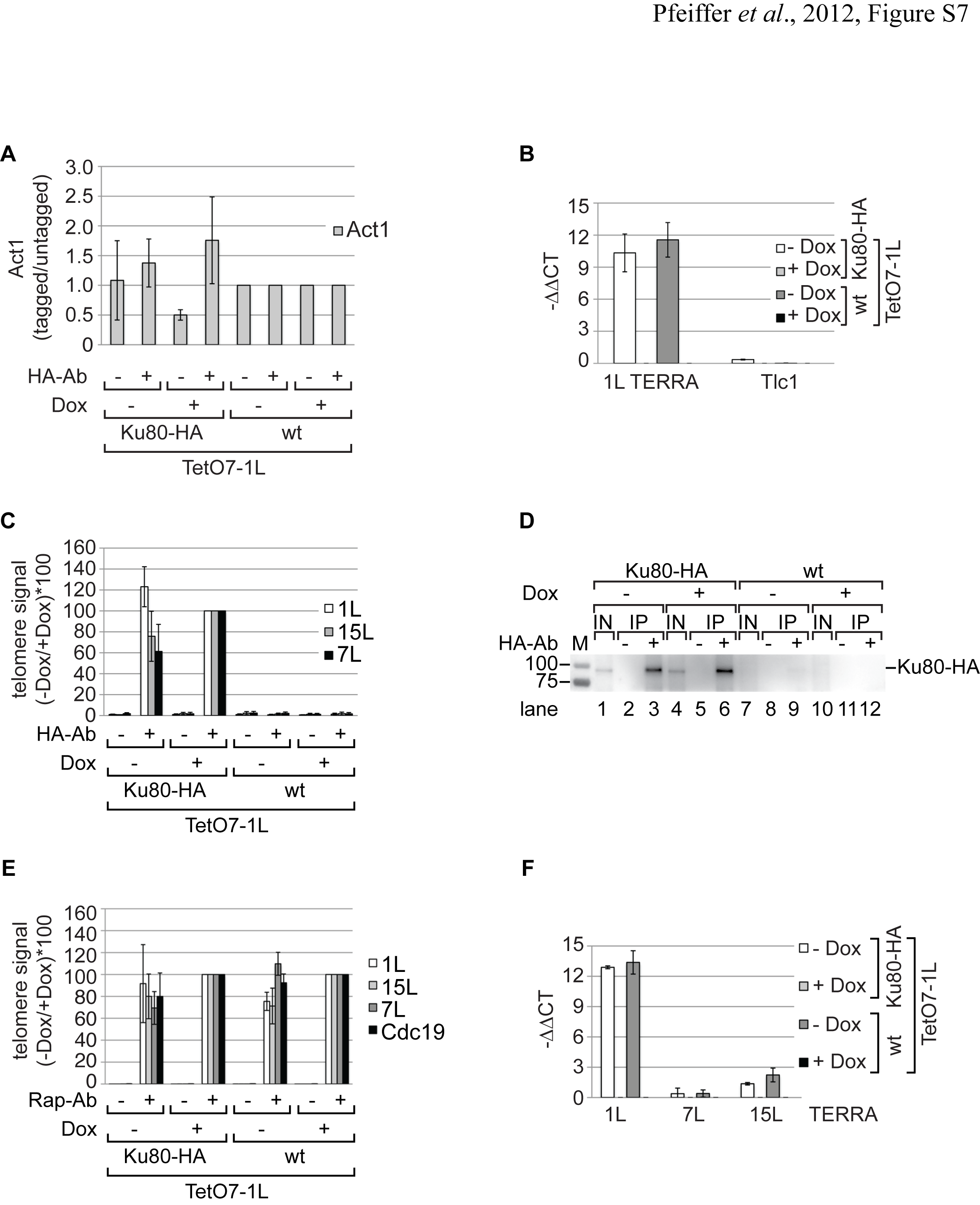

Supplement: Figure S7 — 1L TERRA is specifically pulled down with Ku80-HA and 1L TERRA expression does not interfere with binding of Ku70/80 and Rap1 to telomere 1L. (A) Act1 is not enriched in the HA-tagged fraction in RNA-ChIP experiments using Ku80-HA (see Figure 5A). Yeast strains were grown for 25 generations at 25°C on YPD plates with (+) or without (−) Dox. Ku80-HA associated RNA was immunoprecipitated after an additional growth to exponential phase in rich medium (−/+Dox) at 25°C. A mock IP lacking the HA-antibody as well as an untagged strain (wt) served as controls. The immunoprecipitated Act1 RNA was quantified by real-time PCR and expressed relative to the untagged strain. Values of two independent biological replicates with standard deviation are shown. (B) Quantification of TERRA in samples used for RNA-ChIP of Ku80-HA (see Figure 5A). Yeast strains were grown and RNA was extracted as described in A. Transcribed 1L TERRA and Tlc1 RNA were quantified by qRT-PCR analysis. −ΔΔCT values of strains normalized against actin with standard deviation are shown (two independent biological replicates). The −ΔΔCT values corresponding to each strain grown in +Dox is arbitrarily set to 0. (C) Binding of Ku80 to telomere 1L is not affected by expression of 1L TERRA. Yeast strains were grown for 25 generations at 25°C on YPD plates with (+) or without (−) Dox. Ku80-HA associated chromatin was immunoprecipitated after an additional growth to exponential phase in rich medium (−/+Dox) at 25°C (for details see Materials and Methods). The immunoprecipitated DNA for telomere 1L, 7L, and 15L was quantified by real-time PCR and expressed as percentage of input normalized to each strain under +Dox conditions. A mock IP lacking the HA-antibody as well as an untagged strain served as controls. Values of two independent biological replicates with standard deviation are shown. (D) 1L TERRA expression does not influence the pull-down efficiency of Ku80-HA in ChIP experiments (see C) as determined by Weste [file pgen.1002747.s007.tif]

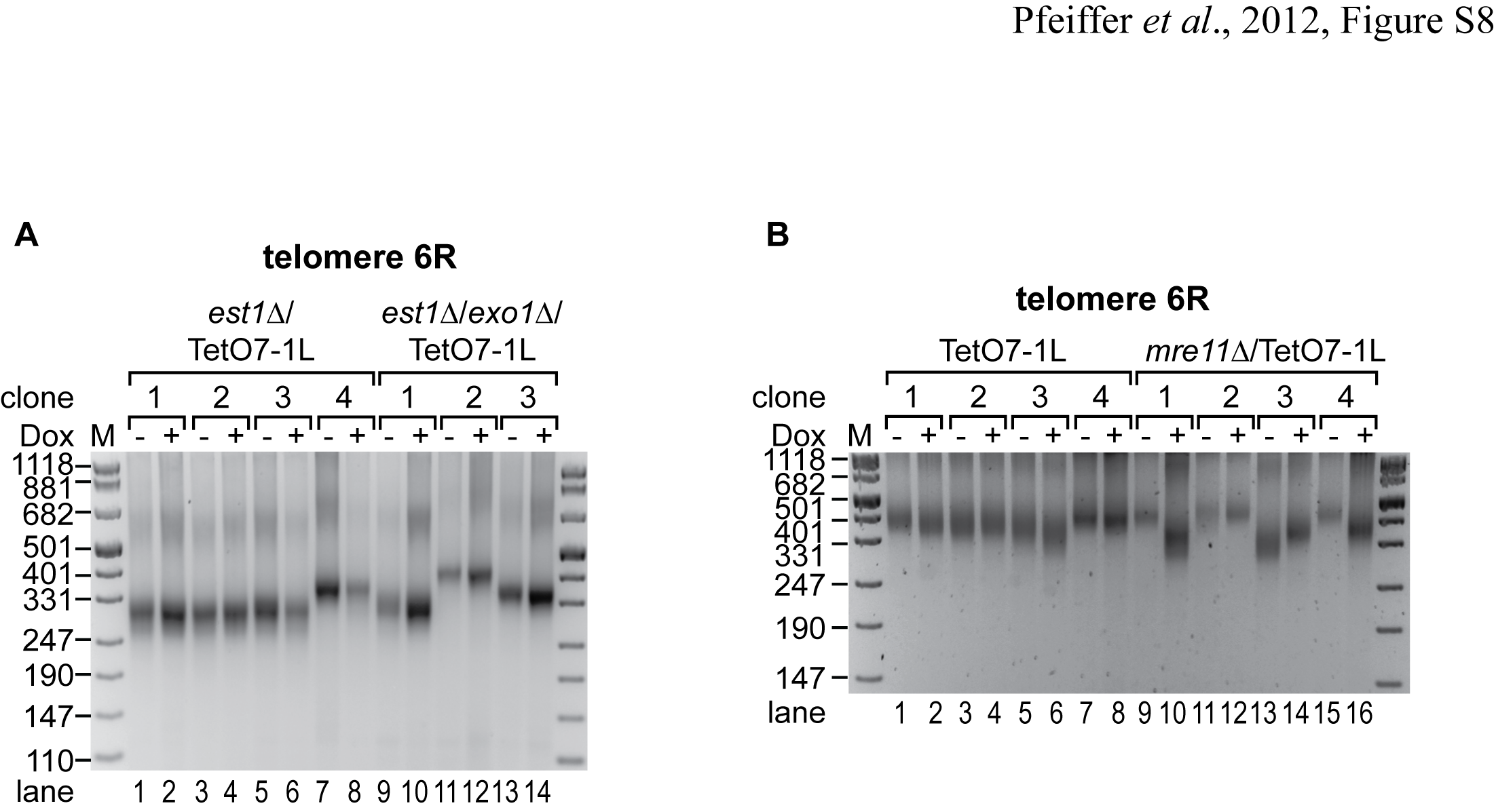

Supplement: Figure S8 — Expression of 1L TERRA does not affect length of telomere 6R in the absence of Exo1, telomerase and Mre11. (A) Length of telomere 6R is not affected upon TERRA expression in the absence of Exo1 and telomerase. est1Δ/TetO7-1L (four independent clones) and est1Δ/exo1Δ/TetO7-1L (three independent clones) strains were grown as described in Figure 6C. Telomere length of 6R was analyzed by telomere PCR on a 2.5% agarose gel. Marker (M) is given in bp. (B) Length of telomere 6R is not affected upon 1L TERRA expression in the absence of Mre11. DNA was extracted from four independent clones of the indicated strains grown as described in Figure 6D. Telomere length at 6R was analyzed by telomere PCR on a 2.5% agarose gel. Marker (M) is given in bp. (TIF) [file pgen.1002747.s008.tif]

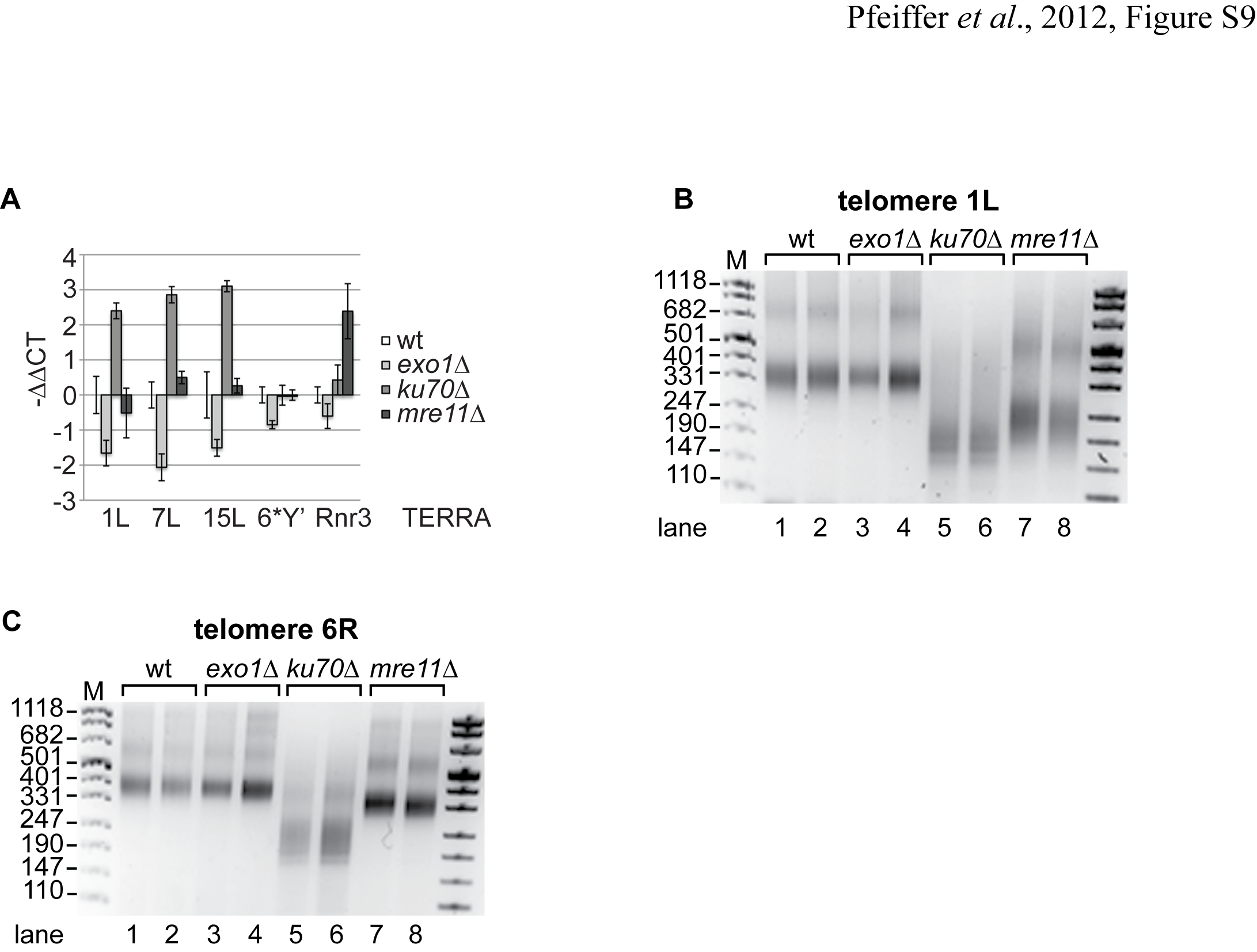

Supplement: Figure S9 — Comparison of TERRA levels in wt, exo1Δ, ku70Δ, mre11Δ. (A) Deletion of Exo1 decreases and deletion of Ku70 increases TERRA levels independent of a DNA damage response (DDR). Deletion of Mre11 (mre11Δ induces a DDR, but has no effect on TERRA levels. qRT-PCR analysis of Rnr3 (checkpoint marker for DDR) and TERRA transcribed from X-only telomeres (1L, 7L, 15L) and from 6 different Y′ telomeres (6*Y′) in wt, exo1Δ, ku70Δ, mre11Δ strains. RNA was extracted from these strains grown in rich medium at 30°C to an OD600 of 0.7. Average −ΔΔCT values of two independent biological replicates normalized against actin with standard deviation are shown. −ΔΔCT values of the wt strain are arbitrarily set to 0. (B) Deletion of KU70 and MRE11 reduces the length of telomere 1L. DNA was extracted from strains grown as described in (A) and analyzed by telomere PCR for telomere 1L on a 2.5% agarose gel. Marker (M) is given in bp. (C) Deletion of KU70 and MRE11 reduces the length of telomere 6R as determined by telomere PCR for 6R. DNA was extracted from strains grown as described in (A). (TIF) [file pgen.1002747.s009.tif]
