# Supplementary material for: TERRA Promotes Telomere Shortening through Exonuclease 1–Mediated Resection of Chromosome Ends
Source: PLoS Genet. 2012 Jun 14;8(6):e1002747. doi: 10.1371/journal.pgen.1002747 (PMC3375253; doi:10.1371/journal.pgen.1002747)
Supplement: Table S1 — Strains used in this study. (PDF) [file pgen.1002747.s010.pdf]

Table S1. Strains used in this study.

| Code | Name | Genotype | Reference/source |
|---|---|---|---|
| YBL7 | wt BY4741 | *MATa his3Δ1 leu2Δ0 ura3Δ0 met15Δ0* | Euroscarf |
| YBL526 | *rat1-1* | *MATa his3Δ1 leu2Δ0 ura3Δ0 met15Δ0 rat1-1::NAT* | Iglesias *et al*., 2011 |
| YNI157 | | *MATa his3Δ1 leu2Δ0 ura3Δ0 met15Δ0 Est2-8G-myc18::TRP1MX6* | Nahid Iglesias, Virginia Zakian |
| YNI322 | *ku70*Δ | *MATa his3Δ1 leu2Δ0 ura3Δ0 met15Δ0 ku70::KAN* | Brian Luke |
| YNI527 | *sir3*Δ | *MATa his3Δ1 leu2Δ0 ura3Δ0 met15Δ0 sir3::HIS3* | Iglesias *et al*., 2011 |
| LVPS-5 | | *MATa his3Δ1 leu2Δ0 ura3Δ0 met15Δ0 Est2-8G-myc18::TRP1MX6 trp1::NAT* | This study |
| LVPS-11 | | *MATa his3Δ1 leu2Δ0 ura3Δ0 met15Δ0 Est2-8G-myc18::TRP1MX6 trp1::NAT ade2::tTA::KAN* | This study |
| LVPS-21 | wt | *MATa his3Δ1 leu2Δ0 ura3Δ0 met15Δ0 Est2-8G-myc18::TRP1MX6 trp1::NAT ade2::tTA* | This study |
| LVPS-31/-33 | TetO7-1L | *MATa his3Δ1 leu2Δ0 ura3Δ0 met15Δ0 Est2-8G-myc18::TRP1MX6 trp1::NAT ade2::tTA URA_ADH1_TetO7_CYC1_1L_TERRA* | This study |
| LVPS-39/-41 | control | *MATa his3Δ1 leu2Δ0 ura3Δ0 met15Δ0 Est2-8G-myc18::TRP1MX6 trp1::NAT ade2::tTA URA_ADH1_ 1L_TERRA* | This study |
| LVPS-118 | *exo1*Δ | *MATa his3Δ1 leu2Δ0 ura3Δ0 met15Δ0 Est2-8G-myc18::TRP1MX6 trp1::NAT ade2::tTA exo1::HIS3MX6* | This study |
| LVPS-123 | *exo1*Δ/TetO7-1L | *MATa his3Δ1 leu2Δ0 ura3Δ0 met15Δ0 Est2-8G-myc18::TRP1MX6 trp1::NAT ade2::tTA URA_ADH1_TetO7_CYC1_1L_TERRA exo1::HIS3MX6* | This study |
| LVPS-131 | *mre11*Δ | *MATa his3Δ1 leu2Δ0 ura3Δ0 met15Δ0 Est2-8G-myc18::TRP1MX6 trp1::NAT ade2::tTA mre11::HIS3MX6* | This study |
| LVPS-132/-133 | *mre11*Δ/TetO7-1L | *MATa his3Δ1 leu2Δ0 ura3Δ0 met15Δ0 Est2-8G-myc18::TRP1MX6 trp1::NAT ade2::tTA URA_ADH1_TetO7_CYC1_1L_TERRA mre11::HIS3MX6* | This study |
| LVPS-137 | *ku70*Δ | *MATa his3Δ1 leu2Δ0 ura3Δ0 met15Δ0 Est2-8G-myc18::TRP1MX6 trp1::NAT ade2::tTA ku70::HIS3MX6* | This study |
| LVPS-138/-139 | *ku70*Δ/TetO7-1L | *MATa his3Δ1 leu2Δ0 ura3Δ0 met15Δ0 Est2-8G-myc18::TRP1MX6 trp1::NAT ade2::tTA URA_ADH1_TetO7_CYC1_1L_TERRA ku70::HIS3MX6* | This study |
| LVPS-151 | Ku80-8G-3HA/TetO7-1L | *MATa his3Δ1 leu2Δ0 ura3Δ0 met15Δ0 Est2-8G-myc18::TRP1MX6 trp1::NAT ade2::tTA URA_ADH1_TetO7_CYC1_1L_TERRA Ku80-8G-3HA::HIS3MX6* | This study |
| | *est1*Δ/TetO7-1L | *MATa his3Δ1 leu2Δ0 ura3Δ0 met15Δ0 Est2-8G-myc18::TRP1MX6 trp1::NAT ade2::tTA URA_ADH1_TetO7_CYC1_1L_TERRA est1::KANMX6* | This study |
| | *est1*Δ/*exo1*Δ/TetO7-1L | *MATa his3Δ1 leu2Δ0 ura3Δ0 met15Δ0 Est2-8G-myc18::TRP1MX6 trp1::NAT ade2::tTA URA_ADH1_TetO7_CYC1_1L_TERRA est1::KANMX6 exo1::HIS3MX6* | This study |
