# Supplementary material for: TERRA Promotes Telomere Shortening through Exonuclease 1–Mediated Resection of Chromosome Ends
Source: PLoS Genet. 2012 Jun 14;8(6):e1002747. doi: 10.1371/journal.pgen.1002747 (PMC3375253; doi:10.1371/journal.pgen.1002747)
Supplement: Table S2 — Oligonucleotides used in this study. (PDF) [file pgen.1002747.s011.pdf]

Table S2. Oligonucleotides used in this study.

| Code | Name | TERRA amplified | Sequence 5'-3' | final concentration in qPCR |
|---|---|---|---|---|
| oBL207 | CA | | CACCACACCCACACACCACACCCACA | |
| oNI20 | TLC1 F | | ACCGTAAATTCTTAAACACTGCTATTGC | 300 nM |
| oNI21 | TLC1 R | | GGTGTGGTGATGGTAGGCTTCC | 300 nM |
| oNI50 | Y' | 8L / 8R / 12L-YP1[a] / 12R-YP2 [a] / 13L / 15R | AGCACTTCTACATAGCCCTAAATAGCC | |
| oNI55 | | | ACCCTGTCCCATTCAACCATAC | |
| oNI62 | Y' 6 F | 8L / 8R / 12L-YP1[a] / 12R-YP2 [a] / 13L / 15R | GGCTTGGAGGAGACGTACATG | 300 nM |
| oNI181 | Y' 6 R | 8L / 8R / 12L-YP1[a] / 12R-YP2 [a] / 13L / 15R | CTCGCTGTCACTCCTTACCCG | 300 nM |
| oNI54 | 1L F | 1L | CGGTGGGTGAGTGGTAGTAAGTAGA | 600 nM |
| oBL1181 | 1L R | 1L | CAGCCCTAATCTAACCCTGG | 600 nM |
| oNI224 | 7L F | 7L | ACGGTTATGATGGGCGGTGGA | 300 nM |
| oNI194 | 7L R | 7L | CTACCCTAACCCTATTCTAACCCAGATC | 300 nM |
| oVP85 | 15L F | 15L | GGGTAACGAGTGGGGAGGTAA | 100 nM |
| oVP86 | 15L R | 15L | CAACACTACCCTAATCTAACCCTGT | 100 nM |
| oVP101 | CDC19 F | | GACATCGGGCTTCCACAATTTCG | 100 nM |
| oVP102 | CDC19 R | | ACAGACATCACACGCCATAGAGG | 100 nM |
| oNI211 | ACT1 F | | GTAACATCGTTATGTCCGGTGGTAC | 300 nM |
| oNI212 | ACT1 R | | CCAAGATAGAACCACCAATCCAGAC | 300 nM |
| oBL284 | RNR3 F | | CCTGATCTTTTCATGAAACG | 600 nM |
| oBL285 | RNR3 R | | CTTTAATTGTTTTACCACGA | 600 nM |
| oVP8 | ADE2 F | | CAATCAAGAAAAACAAGAAAATCGGACAAAACAATCAAGTCACT AGTGGATCTGATATCACCTA | |
| oVP9 | ADE2 R | | ATAATTATTTGCTGTACAAGTATATCAATAAACTTATATACCGCCA GCTGAAGCT | |
| oVP13 | EcoRI-URA3 F | | GTTTTTTGAATTCTGTTAAGCTTATATCAGCACTAACAAACAAAAC AAATACATCAATTCATCATTTTTTTTTTATTC | |
| oVP14 | KpnI-URA3 R | | GTTTTTTGGTACCGGGTAATAACTGATATAATTAAATTGAAG | |

| | | |
|---|---|---|
| oVP15 | AvaI-ADH1 F | GTTTTTTCCCGGGCGCGCCACTTCTAAATAAG |
| oVP16 | BamHI-CYC1 R | GTTTTTTGGATCCCGAATTGATCCGGTAATTTAG |
| oVP22 | TRP1 F | GTGAGTATACGTGATTAAGCACACAAAGGCAGCTTGGAGTCGGATCCCCGGGTTAATTAA |
| oVP23 | TRP1 R | TGCACAAACAATACTTAAATAAATACTACTCAGTAATAACGAATTCGAGCTCGTTTTCGA |
| oVP32 | BamHI-ADH1 R | GTTTTTTGGATCCGATCTGCCGGTAGAGGTG |
| oVP37 | BamHI-1L F | GTTTTTTGGATCCAAGTGAGGGTGAGTATGGCATGTGGTGGTGGTATAAA |
| oVP38 | XbaI-1L R | GTTTTTTTCTAGACAGCCCTAATCTAACCCTGGCCAACCTGTCTCTC |
| oVP39 | 1L-URA3-F | CTGTAGCATCCGTGTGCGTACGTAAAATCAGTATACTCAATTCATCATTTTTTTTTTATTC |
| oVP142 | Exo1 R | GAAAAATATACCTCCGATATGAAACGTGCAGTACTTAACTTGAATTCGAGCTCGTTTAAAC |
| oVP146 | Exo1 F | CATTAAAATAAAAGGAGCTCGAAAAAACTGAAAGGCGTAGAAAGGACGGATCCCCGGGTTAATTAA |
| oKF147 | Est1 F | TATCAGGGGAAAAAGTATATTCCATTAAATGACACATGCCACCATAGATACGGATCCCCGGGTTAATTAA |
| oKF148 | Est1 R | TCATAATATATTTCATATTATGATTTTTTCCCTCACCATTACTTGTTCTCGAATTCGAGCTCGTTTAAAC |
| oVP157 | Mre11 R | GGTTATAAATAGGATATAATATAATATAGGGATCAAGTACAAGAATTCGAGCTCGTTTAAAC |
| oVP161 | Mre11 F | GACGCAAGTTGTACCTGCTCAGATCCGATAAAACTCGACTCGGATCCCCGGGTTAATTAA |
| oVP171 | Ku70 R | TGTATGTAACGTTATAGATATGAAGGATTTCAATCGTCTGAATTCGAGCTCGTTTAAAC |
| oVP173 | Ku70 F | GTTAAGTGACTCTAAGCCTGATTTTAAAACGGGAATATTCGGATCCCCGGGTTAATTAA |
| oVP187 | Ku80 F HA | GTGAACAACACAGTAGGGGAAGTCCAAACAATAGCAATAATGGAGGAGGAGGAGGAGGAGGACGGATCCCCGGGTTAATTAA |
| oVP188 | Ku80 R HA | GTGGTGACGAAAACATAACTCAAAGGATGTTAGACCTTTTGAATTCGAGCTCGTTTAAAC |
| oG18-*BamHI* | | GGATCCGGGGGGGGGGGGGGGGGG |
| oJC43 | | AAATGAGGACTGGGTCATGG |
| oBL1180 | | CCAGGGTTAGATTAGGGCTG |

[a]The left arm of chromosome XII contains two short telomeric Y' elements, 12L-YP1 is more end-proximal and 12L-YP2 is more centromere-proximal.
