# Supplementary material for: TERRA Promotes Telomere Shortening through Exonuclease 1–Mediated Resection of Chromosome Ends
Source: PLoS Genet. 2012 Jun 14;8(6):e1002747. doi: 10.1371/journal.pgen.1002747 (PMC3375253; doi:10.1371/journal.pgen.1002747)
Supplement: Table S3 — Plasmids used in this study. (PDF) [file pgen.1002747.s012.pdf]

Table S3. Plasmids used in this study.

Code	Reference/source
pGEM3ZF	Stratagene
pAG25	Euroscarf
pCM325	Euroscarf
pRS306	Euroscarf
pSH47	Euroscarf
pUG6-tTA	Euroscarf
pSH47	Euroscarf
pFA6a-*HIS3*MX6	Longtine *et al*., 1998
pFA6a-*KAN*MX6	Longtine *et al*., 1998
pFA6a-3HA-*HIS3*MX6	Longtine *et al*., 1998
pLVP001	This study
pLVP002	This study
pLVP009	This study
pLVP010	This study
pLVP011	This study
